# ﻿Revision of Taiwanese and Ryukyuan species of *Pristepyris* Kieffer, 1905, with description of a new species (Hymenoptera, Bethylidae)

**DOI:** 10.3897/zookeys.1102.84953

**Published:** 2022-05-19

**Authors:** Hauchuan Liao, Mamoru Terayama, Katsuyuki Eguchi

**Affiliations:** 1 Department of Biological Sciences, Graduate School of Science, Tokyo Metropolitan University, Minami-Osawa 1-1, Hachioji, Tokyo 192-0397, Japan Tokyo Metropolitan University Tokyo Japan; 2 Department of International Health and Medical Anthropology, Institute of Tropical Medicine, Nagasaki University, Sakamoto 1-12-4, Nagasaki, Nagasaki 852-8523, Japan Nagasaki University Sakamoto Japan

**Keywords:** Flat wasp, Japan, male genitalia, molecular phylogeny, morphology, Pristocerinae, Taiwan

## Abstract

The pristocerine genus *Pristepyris* comprises 38 valid species recorded worldwide, except in the Australian Region. Of them, three species, namely *P.mieae* (Terayama, 1995), *P.tainanensis* (Terayama, 1995) and *P.takasago* (Terayama, 1996), have been recorded from Taiwan and three species, i.e. *P.ishigakiensis* (Yasumatsu, 1955), *P.minutus* (Yasumatsu, 1955) and *P.ryukyuensis* (Terayama, 1999), from the Ryukyus in Japan. In the present study, the species-level classification of both Taiwanese and Ryukyuan species of *Pristepyris* was revised using newly-collected specimens by the external and male genital morphological as well as molecular phylogenetic analysis. Overall, six species of *Pristepyris* were recorded from Taiwan and the Ryukyus. Among these, five were previously recorded for the region and were revised here: *P.ishigakiensis*, *P.mieae*, *P.ryukyuensis*, *P.tainanensis* and *P.zhejiangensis*. Additionally, a new species, *P.seqalu***sp. nov.**, is herein described and illustrated. Furthermore, the species *P.minutus* is transferred to *Eleganesia* and *P.takasago* is synonymized under *P.minutus*. Due to the new combination of *Pristepyrisminutus*, a key to Taiwanese and Ryukyuan species of the genus *Eleganesia*, based on male morphology, is provided in Appendix 1. We confirmed for the first time the correspondence between the male and female species of *P.zhejiangensis* by molecular data. High compatibility in species delimitation patterns, suggested by the morphological and molecular phylogenetic approaches, highlighted the significance of the former approach for accurately classifying aged voucher specimens of Pristocerinae in public collections.

## ﻿Introduction

Bethylidae, also known as flat wasps, are a cosmopolitan family belonging to the Chrysidoidea; they involve approximately 2,900 valid named species (excluding fossil species) that are assigned to 96 genera of nine subfamilies in the current classification (Azevedo et al. 2018; Colombo et al. 2020). Flat wasps are parasitoids, which are potential natural enemies of lepidopteran (Noctuidae, Tortricidae) and coleopteran (Cerambycidae, Curculionidae) pests in farmlands, orchards and timber plantations (Azevedo et al. 2018).

Recently, Alencar et al. (2018) and Azevedo et al. (2018) significantly revised the classification of the subfamily Pristocerinae, by combining conventional morphological examination and molecular phylogenetic analysis. The revised classification is the best working hypothesis for further taxonomic and other related studies in specific geographic regions (Liao et al. 2019, 2021).

As a part of our long-term project to revise and update the species and higher classifications of East and Southeast Asian Bethylidae, we focused on the genus *Pristepyris* (Kieffer 1905; sensu Alencar et al. 2018). This genus consists of 38 validly-named species recorded from the Ethiopian (one species), Nearctic (19 species), Neotropic (three species), Oriental (ten species) and Palaearctic (four species) Regions (Azevedo et al. 2018). Of them, three species, namely *P.mieae*, *P.tainanensis* and *P.takasago*, are recorded from Taiwan and three species, namely *P.ishigakiensis*, *P.minutus* and *P.ryukyuensis*, from the Ryukyu Islands in Japan (Azevedo et al. 2018). In the present study, the species-level classification of Taiwanese and the Ryukyuan species of *Pristepyris* has been revised using an integrative approach of morphological examination and molecular phylogenetic analyses using newly-collected specimens.

## ﻿Materials and methods

### ﻿Sampling sites

**Figure 1.**
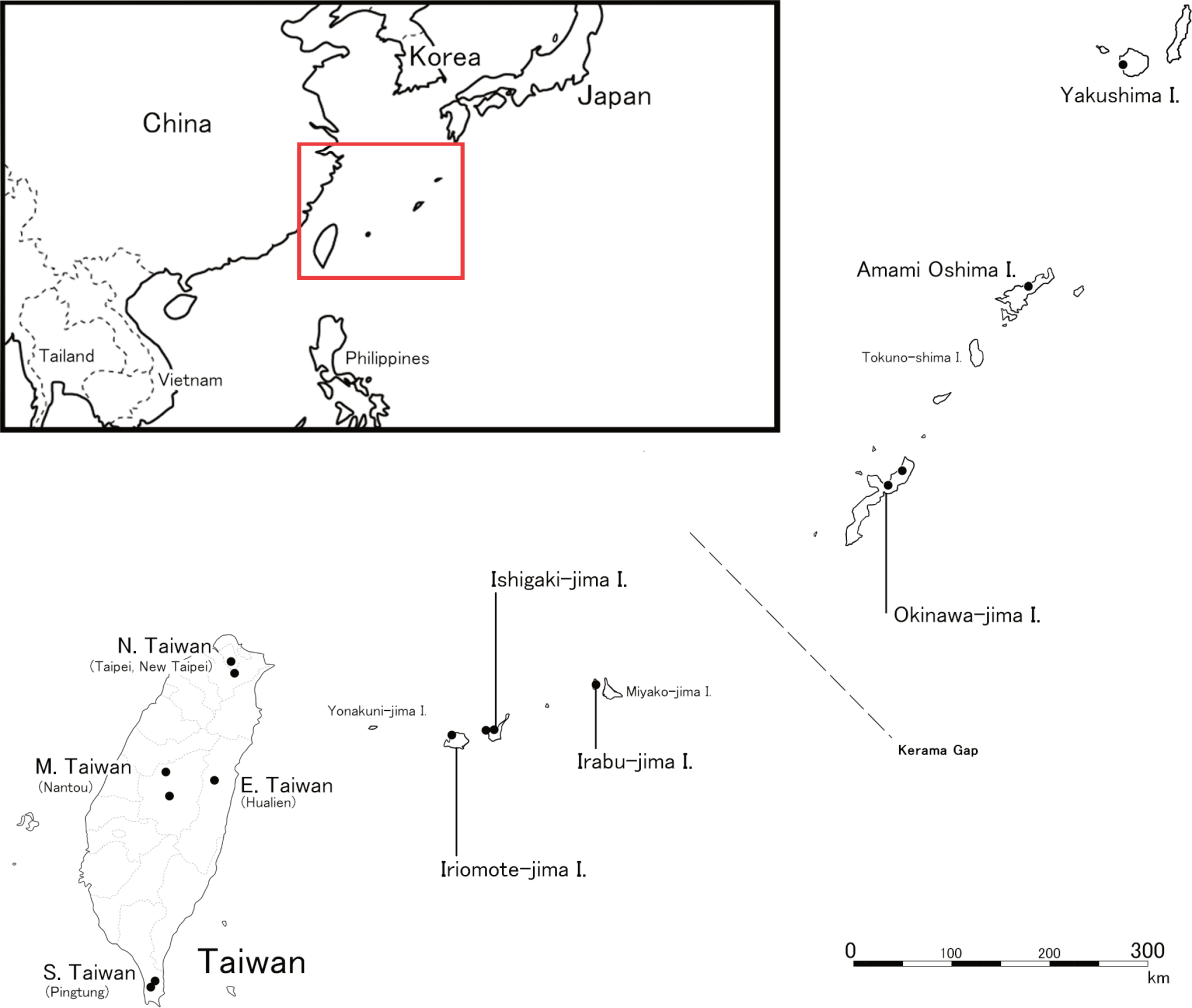
Sampling sites (dots) of *Pristepyris* and *E.minuta* comb. nov. in the present study. From northern Ryukyu to southern Taiwan, including Tokyo City, Japan

*Pristepyris* specimens were collected by sweeping undergrowth along trails in the woody habitats in the following localities: Taipei and New Taipei City (northern Taiwan, Oct 2017, May 2018, Oct 2019); Nantou County (central Taiwan, Mar 2019); Hualian County (eastern Taiwan, Oct 2019); Pingtung County (southern Taiwan, May and Oct 2017); Yakushima, Okinawa Hontou, Irabu–jima, Ishigaki–jima and Iriomote–jima Islands (Aug 2017, Jul 201, Sep 2021); and Tokyo (Apr 2016, Aug 2020) (Fig. 1). Collected specimens were preserved in 99% ethanol.

### ﻿Specimen depository

Specimens examined in the current study are (or will be) deposited in the following institutions:

**HUS**Hokkaido University, Sapporo (Laboratory of Systematic Entomology) (Masahiro Ohara);

**NMNS**National Museum of Natural Science, Taichung City, Taiwan (Jingfu Tsai);

**NIAES–NARO** Institute for Agro-Environmental Sciences–National Agriculture and Food Research Organization, Tsukuba, Japan (Junsuke Yamasako);

**NSMT** National Museum of Nature and Science, Tokyo, Japan (Tsukuba Research Departments, Tsukuba, Japan) (Tatsuya Ide);

**SCAU**South China Agricultural University, China;

**TARI**Taiwan Agricultural Research Institute, Taichung (Chifeng Lee).

### ﻿Morphology-based examination and identification

Following the definition of the genus *Pristepyris* proposed by Alencar et al. (2016) and Azevedo et al. (2018), 76 *Pristepyris* specimens (73 males and three females) were recogniszed in the current study. They were then sorted into morphospecies, based on external and male genital morphology and identified at the species level by referring to the original descriptions or by examining the type materials (or high-resolution images of the type materials provided by NARO) of the following named congeners recorded from Taiwan, the Ryukyus and their adjacent areas (mainland Japan and China).

*P.ishigakiensis* (Yasumatsu, 1955), Japan, original description

*P.japonicus* (Yasumatsu, 1955), Japan, original description. Additional non-type material examined. Two males (JT160420_01, JT200820_03); Minami-osawa, Hachiouji City, Tokyo Pref., Japan, 35°37'11"N, 139°23'03"E, 154 m alt. HauChuan Liao leg. (sweeping); 20/IV/2016, 20/VIII/2020.

*P.mieae* (Terayama, 1995), Taiwan, holotype (female, NARO), examined.

*P.minutus* (Yasumatsu, 1955), Japan, holotype (male, KUF), examined.

*P.rugulosus* (Terayama et al. 2002), China, holotype (male, SCAU), examined.

*P.ryukyuensis* (Terayama, 1999), Japan, holotype (male, NARO), examined.

*P.sinensis* (Terayama et al. 2002), China, holotype (male, SCAU), examined.

*P.tainanensis* (Terayama, 1995), Taiwan, paratype (male, NARO), examined.

*P.takasago* (Terayama, 1995), Taiwan, holotype (male, NARO), examined.

*P.zhejiangensis* (Terayama et al. 2002), China, holotype (male, SCAU), examined.

### ﻿Imaging, measurements, indices and terminology

**Figure 2.**
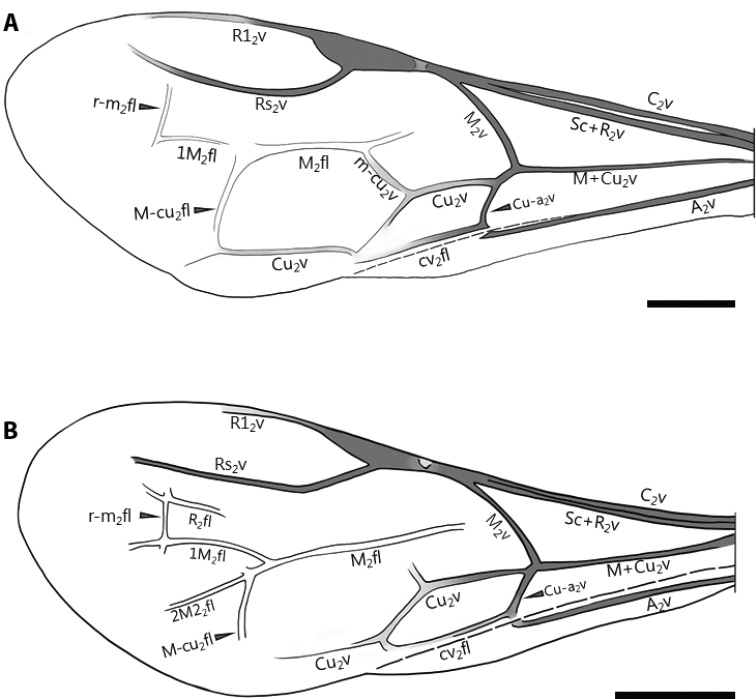
Forewing venation **A***Pristepyris*, drawing was made based on *P.ishigakiensis* (JI170808_38). **B***Eleganesia*, drawing was made, based on *E.minuta* comb. nov. (JO190717_13). Scale bars: 0.5 mm.

Morphological examination, imaging, line-drawing and measurement were performed as in Liao et al. (2021): **HL**, head length, from the anterior margin of the clypeus to the posterior margin of the head in dorsal view; **HW**, maximum width of head including compound eyes; **EL**, compound eye length in dorsal view; **POL**, minimum distance between median margins of posterior ocelli; **WOT**, maximum distance between lateral margins of posterior ocelli; **AOL**, minimum distance between antero-inner margin of posterior ocellus and posterolateral margin of anterior ocellus; **OOL**, minimum distance between anterolateral margin of posterior ocellus and posteromedian margin of compound eye; **DAO**, transverse diameter of anterior ocellus; **LM**, length of mesosoma, measured from the anteriormost flange of the pronotum to the posteriormost of the metapectal-propodeal complex; **LMT**, length of metasoma, measured from the posteriormost of the metapectal-propodeal complex to the apex of the gaster (excluding the sting); **LPD**, length of the dorsal pronotal area, measured in lateral view from the junction between the pronotal flange and dorsal pronotal area to the posteriormost point of the dorsal pronotal area; **WPD**, maximum width of the dorsal pronotal area; **LP**, length of the metapectal-propodeal complex, measured from the junction of the transverse anterior carina and median carina to the posteriormost of the metapectal-propodeal complex; **WP**, width of the metapectal-propodeal complex, measured along the transverse line passing the anteriormost points of the propodeal spiracles; **TL**, total body length. Sensilla placodea (antennal plate organs) were taken by a JSM-6510 scanning electron microscope. The morphological terminology follows predominantly Lanes et al. (2020). Fig. 2 depicts the abbreviations of forewing veins.

### ﻿DNA sequencing

A total of 39 specimens of the ingroup morphospecies, including *P.ishigakiensis*, *P.japonicus*, *P.minutus* and *P.zhejiangensis*, were studied for their molecular phylogenetic analyses, together with 67 specimens of 45 outgroup morphospecies of the subfamilies Pristocerinae (12 genera), Epyrinae (one genus) and Scleroderminae (one genus) (Table 1). DNA was extracted from the second and third right legs of each specimen using the Chelex-TE-ProK protocol (Satria et al. 2015) with an incubation time of 24 hours. Nuclear 28S ribosomal RNA (rDNA) was amplified and sequenced using the primer D2B (GTCGGGTTGCTTGAGAGTGC) and D3Ar (TCCGTGTTTCAAGACGGGTC) (Fisher and Smith 2008). Mitochondrial cytochrome oxidase subunit 1 (COI) genes were amplified and sequenced using the primer LCO1490 (GGTCAACAAATCATAAAGATATTGG) and HCO2198 (ATGTGCGTTCRAAATGTCGATGTTCA) (Folmer et al. 1994). Polymerase chain reaction (PCR) amplification, cycle sequencing reactions, sequencing using ABI PRISM 3130xl (Applied Biosystems) and sequence assembly using ChromasPro 1.7.6 (Technelysium Pty Ltd., Tewantin QLD, Australia) were conducted by following Satria et al. (2015). COI was aligned by ClustalW (Thompson et al. 1994) built-in MEGA X (Kumar et al. 2018) and 28S sequencing done using Multiple Sequence Alignment Software, MAFFT version 7 (Katoh et al. 2019, https://mafft.cbrc.jp/alignment/software/).

**Table 1.** Data of specimens used for molecular phylogenetic analysis. The data of the morphospecies name labelled “IA” were taken by Alencar et al. (2018). AU = Australia, BR = Brazil, JP = Japan, KY = Kenya, MA = Madagascar,NI = Nigeria, PNG = Papua New Guinea, TH = Thailand, TW = Taiwan.

Specimen no.	Country	Morphospecies	Sex	Coordinates	Accession number	
					28S	COI
***Pristepyris***						
JI170808_30	JP	*P.ishigakiensis*	M	24°26'N, 124°05'E	LC705070	LC704953
JI170808_33	JP	*P.ishigakiensis*	M	24°26'N, 124°05'E	LC705067	LC704954
JI170808_34	JP	*P.ishigakiensis*	M	24°26'N, 124°05'E	LC705068	LC704955
JI170808_36	JP	*P.ishigakiensis*	M	24°26'N, 124°05'E	LC705069	LC704956
TP171019_10	TW	*P.ishigakiensis*	M	24°04'N, 120°46'E	LC705075	LC704961
TH171007_37	TW	*P.ishigakiensis*	M	23°49'N, 121°33'E	LC705071	LC704957
TH171007_40	TW	*P.ishigakiensis*	M	23°49'N, 121°33'E	LC705072	LC704958
TH171007_41	TW	*P.ishigakiensis*	M	23°49'N, 121°33'E	LC705073	LC704959
TH171007_42	TW	*P.ishigakiensis*	M	23°49'N, 121°33'E	LC705074	LC704960
JT200820_03	JP	*P.japonicus*	M	26°34'N, 128°00'E	LC705077	LC704963
TP170606_13	TW	*P.seqalu* sp. nov.	M	22°08'N, 120°48'E	LC705062	LC704964
TP170606_26	TW	*P.seqalu* sp. nov.	M	22°08'N, 120°48'E	LC705063	LC704949
TN170427_01	TW	*P.zhejiangenisis*	F		LC705084	LC704971
TNT171019_04	TW	*P.zhejiangenisis*	M	24°51'N, 121°33'E	LC706441	LC704972
TNT180504_01	TW	*P.zhejiangenisis*	M	24°51'N, 121°33'E	LC491436	LC490571
TN190315_24	TW	*P.zhejiangenisis*	M	23°51'N, 120°56'E	LC704973	LC704973
JM190717_31	JP	*P.zhejiangenisis*	M	24°49'N, 125°13'E	LC705070	LC704966
JM190717_32	JP	*P.zhejiangenisis*	M	24°49'N, 125°13'E	LC705067	LC704967
JM190717_37	JP	*P.zhejiangenisis*	M	24°49'N, 125°13'E	LC705082	LC704969
JM190717_38	JP	*P.zhejiangenisis*	M	24°49'N, 125°13'E	LC705083	LC704970
JIR190717_47	JP	*P.zhejiangenisis*	M	24°23'N, 123°48'E	LC705078	LC704965
	TH	*P.* sp. 2 (IA)	M		MG760740	MG760791
	TH	*P.* sp. 3 (IA)	M		MG760739	MG760790
***Acrenesia***						
	BR	*A.* sp. 10 (IA)	M		MG760753	MG760804
	BR	*A.* sp. 11 (IA)	M		MG760754	MG760805
	BR	*A.* sp. 12 (IA)	M		MG760755	MG760806
	BR	*A.* sp. 13 (IA)	M		MG760756	MG760807
	BR	*A.* sp. 14 (IA)	M		MG760757	MG760808
***Apenesia***						
JO180202_01	JP	*A.makiharai*	F		LC598842	LC598798
JK171031_03	JP	*A.makiharai*	F		LC705058	LC704945
JK171031_04	JP	*A.makiharai*	F		LC705059	LC704946
	BR	*A.perlonga* (IA)	M		MG760761	MG760812
	PNG	*A.* sp. 1 (IA)	M		MG760759	MG760810
	PNG	*A.* sp. 2 (IA)	M		MG760760	MG760811
***Austranesia***						
	AU	*A.* sp. 16 (IA)	M		MG760750	MG760801
	AU	*A.* sp. 17 (IA)	M		MG760751	MG760802
	AU	*A.* sp. 18 (IA)	M		MG760752	MG760803
***Cleistepyris***						
	BR	*C.* sp. 1 (IA)	M		MG760774	MG760830
	BR	*C.* sp. 2 (IA)	M		MG760776	MG760832
	BR	*C.* sp. 3 (IA)	M		MG760780	MG760836
***Dissomphalus***						
TP170606_28	TW	*D.wusheanus*	M	22°08'N, 120°48'E	LC704947	LC704947
TP170606_30	TW	*D.wusheanus*	M	22°08'N, 120°48'E	LC704950	LC704950
	NI	*D.* sp. 2 (IA)	M		MG760768	MG760821
	NI	*D.* sp. 3 (IA)	M		MG760769	MG760822
***Dracunesia***						
	BR	*D.* sp. 19 (IA)	M		MG760747	MG760798
	BR	*D.* sp. 21 (IA)	M		MG760748	MG760799
	BR	*D.* sp. 22 (IA)	M		MG760749	MG760800
***Eleganesia***						
TN160725_25	TW	*E.chitouensis*	M	24°05'N, 121°01'E	LC598843	LC598799
TP170606_25	TW	*E.chitouensis*	M	22°07'N, 120°47'E	LC598846	LC598800
TN181022_01	TW	*E.meifuiae*	M	24°05'N, 121°10'E	LC598862	LC598807
JO170808_05	JP	*E.minuta* comb. nov	M	26°34'N, 128°00'E	LC705098	LC704986
JA170808_13	JP	*E.minuta* comb. nov	M	28°16'N, 129°19'E	LC705092	LC704980
JI170808_28	JP	*E.minuta* comb. nov	M	24°26'N, 124°05'E	LC705093	LC704981
JI170808_35	JP	*E.minuta* comb. nov	M	24°26'N, 124°05'E	LC705099	LC704987
TNT180629_11	TW	*E.minuta* comb. nov	M	24°54'N, 121°30'E	LC705103	LC704991
TNT180706_01	TW	*E.minuta* comb. nov	M	24°53'N, 121°34'E	LC705104	LC704992
TNT180706_06	TW	*E.minuta* comb. nov	M	24°53'N, 121°34'E	LC705105	LC704993
TNT180706_07	TW	*E.minuta* comb. nov	M	24°53'N, 121°34'E	LC705106	LC704994
TNT180706_08	TW	*E.minuta* comb. nov	M	24°53'N, 121°34'E	LC705107	LC704995
TN181022_47	TW	*E.minuta* comb. nov	M	23°51'N, 120°56'E	LC705102	LC704990
JO190717_15	JP	*E.minuta* comb. nov	M	26°45'N, 128°12'E	LC705100	LC704988
JIR190717_49	JP	*E.minuta* comb. nov	M	24°23'N, 123°48'E	LC704985	LC704985
JIR190717_54	JP	*E.minuta* comb. nov	M	24°23'N, 123°48'E	LC705094	LC704982
JT200820_05	JP	*E.minuta* comb. nov	M	26°34'N, 128°00'E	LC705101	LC704989
JK210921_05	JP	*E.minuta* comb. nov	M	30°18'N, 130°25'E	LC705095	LC704983
JK210921_07	JP	*E.minuta* comb. nov	M	30°18'N, 130°25'E	LC705096	LC704984
TN190315_26	TW	*E.takasago*	M	23°52'N, 120°55'E	LC598834	LC598874
TP170606_C2	TW	*E.takasago*	F	22°07'N, 120°48'E	LC598838	LC598876
TT191007_09	TW	*E.takasago*	M	25°05'N, 121°32'E	LC598839	LC598877
JT200820_02	TW	*E.elegans*	M	35°37'N, 139°23'E	LC598803	LC598857
JM190717_46	JP	*E.kijimuna*	M	24°55'N, 125°18'E	LC598819	LC598848
JO170808_04	JP	*E.kijimuna*	M	26°34'N, 128°00'E	LC598820	LC598849
TP170606_14	TW	*E.paiwan*	M	22°07'N, 120°48'E	LC598818	LC598859
***Epynesia***						
JO190717_22	JP	*E.bishamon*	M	26°45'N, 128°12'E	LC598841	LC598879
TN170110_27	TW	*E.bishamon*	M		LC704952	LC704952
TD200628_01	TW	*E.bishamon*	F		LC704951	LC704951
***Pristocera***						
TH191007_25	Taiwan	*P.formosana*	M	23°56'N, 121°31'E	LC705061	LC704948
TNT171019_01	Taiwan	*P.formosana*	M	24°51'N, 121°33'E	LC705087	LC704975
TP171019_08	TW	*P.formosana*	M	22°07'N, 120°45'E	LC490570	LC490572
	KY	*P.* sp. 1 (IA)	M		MG760741	MG760792
	UAE	*P.* sp. 2 (IA)	M		MG760772	MG760825
	TH	*P.* sp. 3 (IA)	M		MG760770	MG760823
	KY	*P.* sp. 4 (IA)	M		MG760742	MG760793
***Propristocera***						
JO170808_01	JP	*P.okinawensis*	M	26°34'N, 128°00'E	LC479553	LC480272
TN160725_9-2	TW	*P.okinawensis*	M	24°05'N, 121°02'E	LC479556	LC480275
TP170606_18	TW	*P.okinawensis*	M	22°07'N, 121°02'E	LC479561	LC480280
JI170808_19	JP	*P.seediq*	M	24°26'N, 124°05'E	LC479571	LC480290
TNT180706_02	TW	*P.seediq*	M	24°52'N, 121°34'E	LC479579	LC480298
TN160725_01	TW	*P.seediq*	M	24°05'N, 121°02'E	LC479576	LC480295
TP170606_32	TW	*P.seediq*	M	22°07'N, 120°47'E	LC479582	LC480301
JA170808_14	JP	*P.pingtungensis*	M	28°16'N, 129°19'E	LC479543	LC480262
JI170808_17	JP	*P.pingtungensis*	M	24°26'N, 124°05'E	LC479544	LC480263
TH191007_38	TW	*P.pingtungensis*	M	24°01'N, 121°32'E	LC705088	LC704976
TN160725_7-2	TW	*P.pingtungensis*	M	24°05'N, 121°02'E	LC479546	LC480265
TP171019_15	TW	*P.pingtungensis*	M	22°07'N, 120°45'E	LC479552	LC480271
***Protisobrachium***						
	MA	*P.* sp. 2	M		MG760767	MG760820
***Pseudisobrachium***						
JI170808_27	JP	*P.ryukyunum*	M	24°26'N, 124°06'E	LC705091	LC704977
TN170110_22	TW	*P.ryukyunum*	M	24°02'N, 121°10'E	LC705090	LC704978
TT191007_01	TW	*P.ryukyunum*	M	25°05'N, 121°36'E	LC705089	LC704979
	BR	*P.* sp. 1 (IA)	M		MG760787	MG760843
	BR	*P.* sp. 2 (IA)	F		MG760788	MG760844
	USA	*P.* sp. 3 (IA)	M		MG760789	MG760845
***Trichiscus***						
	KY	*T.* sp. 1 (IA)	M		MG760764	MG760817
	KY	*T.* sp. 2 (IA)	M		MG760765	MG760818
***Holepyris***						
JT200820_11	JP	*H.benten*	M	26°34'N, 128°00'E	LC705108	LC704996
***Sclerodermus***						
JK171103_01	JP	*Sclerodermus* sp.	F		LC705109	LC704997

### ﻿Molecular phylogenetic analyses and calculation of genetic distances

Maximum Likelihood (ML) analysis was performed for the concatenated dataset of the COI and 28S datasets (hereafter referred to as the COI + 28S dataset) using IQ tree; ultrafast bootstrap (UFB; Minh et al. 2013) and SH-aLRT (Guindon et al. 2010). Prior to the ML analysis, the model TIM3 + F + G4 was selected for the 28S dataset (472-bp) and TPM2 + F + I + G4 for the COI dataset (602-bp) using ModelFinder and were run using partition analysis in iqtree-2.1.1 (Minh et al. 2020; http://www.iqtree.org/) under the Bayesian Information Criterion (BIC). Furthermore, support values were determined from 1,000 re-samplings.

Bayesian Inference (BI) analyses were performed for the COI + 28S dataset using ExaBayes version 1.4 (Aberer et al. 2014) under the default substitution model GTR+G for 10,000,000 generations. The trees were sampled for every 500 generations, tuning parameters every 100 generations and the first 25% of the trees were discarded as burn-in. Tracer version 1.7.1 (Rambaut et al. 2018; http://tree.bio.ed.ac.uk/software/tracer/) was used for checking steady states of all parameter values of the runs. The posterior probability densities were similar between the runs and the effective sample size of parameter values was > 200. A final BI tree was generated using TreeAnnotator 1.8.4 (Drummond et al. 2012). The ML trees were displayed using Figtree 1.4.3 (http://tree.bio.ed.ac.uk/software/figtree/) and edited using FireAlpaca 5.5.1.

Pairwise p-distances and Kimura two-parameter (K2P) distances were calculated for the 28S and COI datasets using MEGA7 (Tamura et al. 2013).

## ﻿Results

### ﻿Morphospecies recognition based on the male

A total of 73 male specimens of *Pristepyris* were assigned into four named species and a novel species, i.e. *P.ishigakiensis*, *P.japonicus*, *P.minutus*, *P.zhejiangensis* and *P.seqalu* sp. nov. The details of the morphological features are provided in the taxonomy section.

The type material (holotype only) of *Pristepyrisryukyuensis* lacks its metasoma. The morphological information of the male genitalia, which is indispensable for discriminating similar species, according to the general external morphology (Liao et al. 2019, 2021) was unavailable. Therefore, the present study could not provide any evidence to support or reject the discrimination between *P.ryukyuensis* and *P.tainanensis* and between *P.ryukyuensis* and *P.zhejiangensis*. Furthermore, no metasomal and genital morphologies have been described in the original description of *P.tainanesis*; hence, we did not have an opportunity to dissect and examine the male genitalia to determine the type material, so we tentatively treated *P.ryukyuensis* and *P.tainanensis* as different species. These obscurities in species discrimination will be solved when many specimens from the Ryukyus, Taiwan and the eastern coastal region of mainland China become available for integrative taxonomy in the future.

*Pristepyrisminutus* was morphologically characterized by the following features of the male genitalia and was well distinguished from four other named species of *Pristepyris* recognized above and *Pristepyrisrugicollis* (Kieffer, 1905); type species of *Pristepyris*, morphological information were obtained from Azevedo and Alencar (2009); gonostipes fused to harpe in dorsal portion in *P.minutus* and fully divided from harpe in the other four species and *P.rugicollis*; aedeagus with unrecognized apical lobe and with enlarged ventral and dorsal valves in *P.minutus* and distinctly elongated apical lobe in the other four species and *P.rugicollis*.

Summarising the results of the morphological examination, the five male-based species of *Pristepyris* were assigned into two groups: group A consisting of *P.ishigakiensis*, *P.japonicus*, *P.zhejiangensis*, *P.seqalu* sp. nov. and *P.rugicollis*; group B had *P.minutus* based on the male genital morphology.

### ﻿Molecular phylogenetic analyses and DNA barcoding

Molecular phylogenetic analyses recovered 15 major clades (including a far distant lineage, i.e. *Pseudisobrachium*) with higher support values (UFB ≥ 95/SH-aLRT ≥ 80/pp ≥ 0.95) and longer basal branches, which were almost consistent with the boundaries of genera proposed previously (Alencar et al. 2018; Azevedo et al. 2018). However, *Pristepyris* (sensu Alencar et al. 2018) was recorded as a polyphyletic group comprising the following two phylogenetically far distant clades with high support values: Clade α (100/100/0.98) involving four male-based species of the group A; Clade β (100/100/1) consisting solely of *P.minutus* (group B) and exhibiting the sister relationship with the clade consisting of six Taiwanese and Ryukyuan *Eleganesia* with strong support values (98/93/1). Similarly, *Acrenesia* was also recorded as a polyphyletic group consisting of two phylogenetically far distant clades with strong support values (100/100/1 in the Clade γ and δ, respectively). However, because the Clade γ and δ of *Acrenesia* remain undetected from Taiwan and the Ryukyus, this issue will be resolved in a separate study in the future.

Fourteen of the 15 distinct clades within the subfamily Pristocerinae were further grouped into two higher clades: Clade I (72.8/92/1) consisting of *Dissomphalus*, *Protisobrachium*, *Trichicus*, *Pristepyris*, *Pristocera* and *Propristocera*; Clade II (88.3/91/0.96) consisting of *Apenesia*, “*Acrenesia*” (Clade γ, δ), *Epynesia*, *Cleistepyris*, *Eleganesia* (including *P.minutus*), *Austranesia* and *Dracunesia*. The position of *Pseudisobrachium* remained unclear due to suspected long-branch attraction from the phylogenetic trees.

Two female specimens were assigned by DNA barcoding into *P.zhejiangensis*. The maximum distance within each of the species was remarkably smaller than the minimum distance in all pairs of the species, i.e. the DNA barcode gap was distinct (Table 2). In the Clade α, the maximum intraspecific p-distances calculated, based on the 28S dataset, were 0.2% for *P.ishigakiensis*, 0% for *P.seqalu* sp. nov. and 0% for *P.zhejiangensis*; however, the minimal interspecific p-distances calculated, based on the 28S datasets, ranged from 0.6%–5.2%. The maximum intraspecific p-distances calculated, based on the COI dataset, were 6.5% for *P.ishigakiensis*, 0% for *P.seqalu* sp. nov. and 8.4% for *P.zhejiangensis*, whereas the minimal interspecific p-distances calculated, based on the COI dataset, ranged from 11.7% to 14.1%.

**Table 2.** The minimal interspecific distances calculated, based on the 28S and COI sequence datasets. Upper diagonal shows the distance in K2P model and lower diagonal shows the distance in p distance (%). N, number of specimens; max p, maximum p distance within the species; max K2P, maximum distance in K2P model within the species.

Datasets and Species	1	2	3	4
28S				
1. *P.ishigakiensis*		0.006	0.054	0.011
(N = 9; max p = 0.2; max K2P = 0.002)				
2. *P.japonicus*	0.6		0.056	0.010
(N = 1)				
3. *P.seqalu* sp. nov.	5.1	5.2		0.047
(N = 2; max p = 0; max K2P = 0)				
4. *P.zhejiangensis*	1.1	0.9	4.5	
(N = 9; max p = 0; max K2P = 0)				
COI				
1. *P.ishigakiensis*		0.156	0.161	0.128
(N = 9; max p = 6.5; max K2P = 0.069)				
2. *P.japonicus*	13.7		0.155	0.132
(N = 1)				
3. *P.seqalu* sp. nov.	14.1	13.7		0.130
(N = 2; max p = 0; max K2P = 0)				
4. *P.zhejiangensis*	11.8	12.3	11.7	
(N = 9; max p = 8.4; max K2P = 0.092)				

## ﻿Discussion

### ﻿Confirmation of the species boundaries in *Pristepyris*

**Figure 3.**
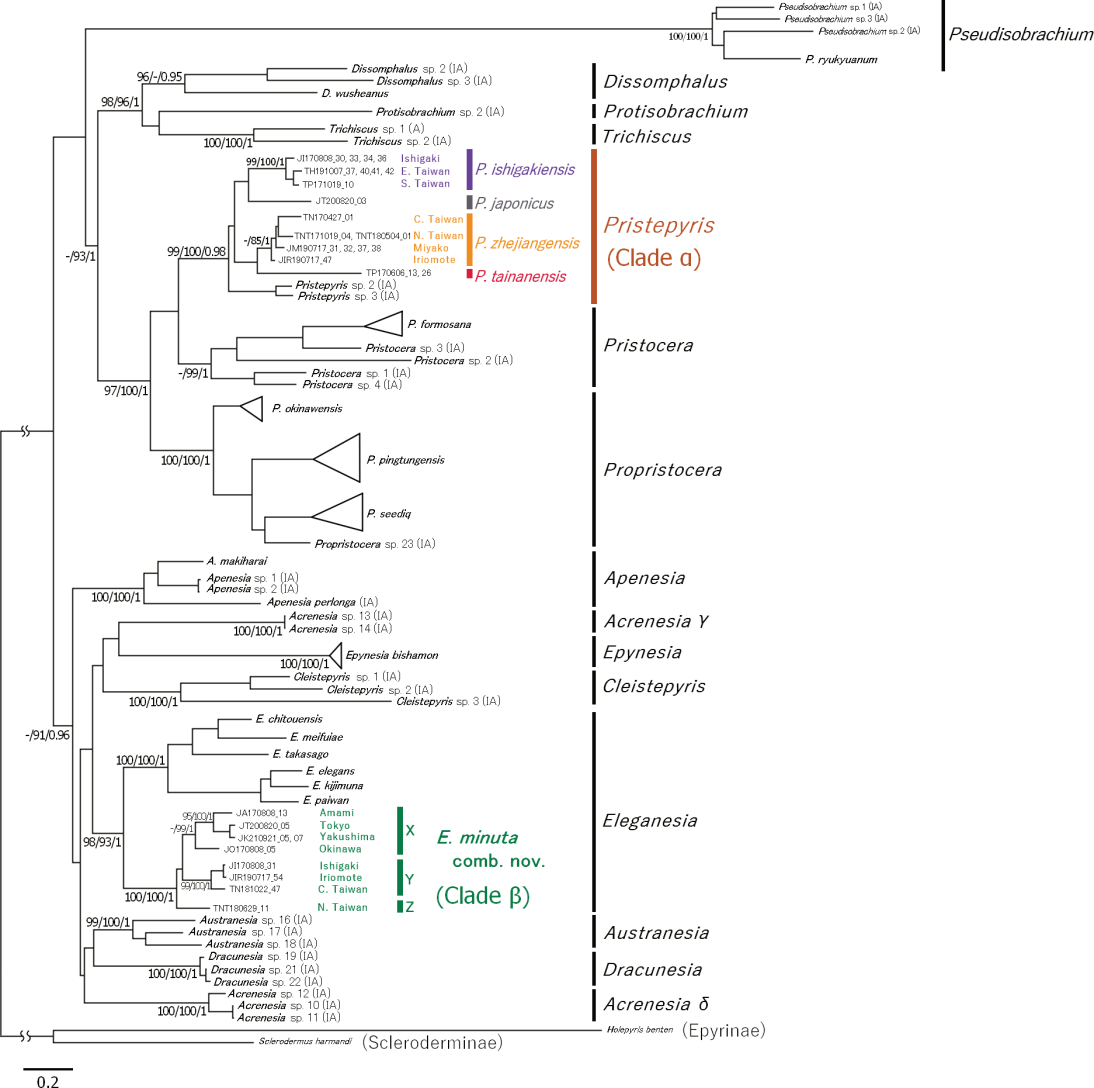
ML tree based on the 28S + COI dataset (1,075 bp in length). Ultrafast bootstrap (UFB), SH-aLRT and posterior probability (pp) values are given beside the nodes. The values were omitted when UFB < 95, SH-aLRT < 80 and pp < 0.90. Tips are labelled with specimen ID. **Tokyo**, Tokyo Metropolis; **Yakushima**, Yakushima Island; **Amami**, Amami-Oshima Island; **Okinawa**, Okinawa-Hontou Island; **Irabu**, Irabu–jima Island; **N. Taiwan**, Northern Taiwan; **C. Taiwan**, Central Taiwan; **E. Taiwan**, Eastern Taiwan; **S. Taiwan**, Southern Taiwan.

Each of the four species of group A and the one species of group B were recovered as an independent lineage in the molecular phylogenetic analyses (Fig. 3). Both the 28S and COI datasets of group A showed distinct DNA barcode gaps in all pairs of the species. Therefore, five different species can be consistently recognized.

Group A (the Clade α nested in the Clade I) and *Pristepyrisrugicollis* (the type species of the genus) exhibited significant similarity in the male genital morphology; therefore, it could reasonably be determined as *Pristepyris* sensu stricto. This implies that *Pristepyrisminutus* (the Clade β nested in the Clade II) is independent at the genus level from *Pristepyris*. Therefore, “*P.minutus*” is herein assigned to *Eleganesia* (new combination). The formal taxonomic treatment is shown in the taxonomy section.

Pristocerine genera can be assigned to two higher groups, based on the morphology of male genitalia. The complete articulation between gonostipes and harpe was observed in the group P: *Apenesia*, *Dissomphalus*, *Epynesia*, *Pristepyris* sensu stricto, *Pristocera* and *Propristocera* in our study similar to earlier studies (Alencar et al. 2018; Azevedo et al. 2018; Liao et al. 2019; Alencar and Azevedo 2020). Contrastingly, the incomplete articulation or complete fusion between gonostipes and harpe was observed in group Q: *Austranesia*, *Eleganesia* and *Epynesia* (Alencar et al. 2018; Liao et al. 2021). The division between P and Q groups seems to be supported by the antennal micromorphology of the female wasps (Fig. 19). Our examination of antennal structure with limited taxa revealed that the sensilla placodea are narrow on the antennae and long in *Pristepyris* sensu stricto and *Propristocera* of group P (Fig. 19C, D) and round in *Eleganesia* and *Apenesia* of group Q (Fig. 19A, B). Groups P and Q correspond to Clade I and II, respectively. Furthermore, *Caloapenesia*, *Calobrachium* and *Pseudisobrachium* show huge gonostipes and unique apically-divided harpe in the subfamily (Azevedo 2008; Gobbi and Azevedo 2010, 2014, 2016). *Pseudisobrachium* was recovered to be independent of both Clade I and II in phylogenetic trees. Therefore, it is likely that the phylogenetic and morphological examination with the further comprehensive taxon sampling will recognize multiple suprageneric taxa (tribes) within the subfamily Pristocerinae.

### ﻿Geographic genetic divergence observed in *Eleganesiaminuta* comb. nov.

*Eleganesiaminuta* comb. nov. is widespread, but genetically subdivided into three COI lineages: X from the Kanto area of Japan to Okinawa Hontou Island; Y from Ishigaki–jima Island to Taiwan; and Z from Taiwan. The p-distance among the COI sublineages ranged between 11.1% and 14.6%. By referring to the molecular clock in COI of insects (Papadopoulou et al. 2010), which shows 3.36%–3.54% divergence per one million years, we can estimate that the COI sublineages have diverged for approximately three to four million years. Females of *E.minuta* comb. nov. are apterous; hence, they can be distributed only over long distances by phoretic copulation (Gordh 1990; Azevedo et al. 2016) or ocean currents that carry rotten logs in which fertile female wasps or parasitized hosts hide. This phenomenon can reduce female-mediated gene flow, thereby causing clear genetic divergence of the COI gene, which is inherited maternally.

### ﻿Taxonomy of the Taiwanese and Ryukyuan species of *Pristepyris* and *Eleganesia*

**Bethylidae Forster, 1856**

**Pristocerinae Mocsary, 1881**

#### *Pristepyris* Kieffer, 1905

##### ﻿ Pristepyris seqalu sp. nov.

Taxon classificationAnimaliaHymenopteraBethylidae

1879051D-033A-56D4-B595-AB0A8E105E36

http://zoobank.org/773D354A-5DF3-45A1-BCF6-3182C8D6A161

**Figure 4.**
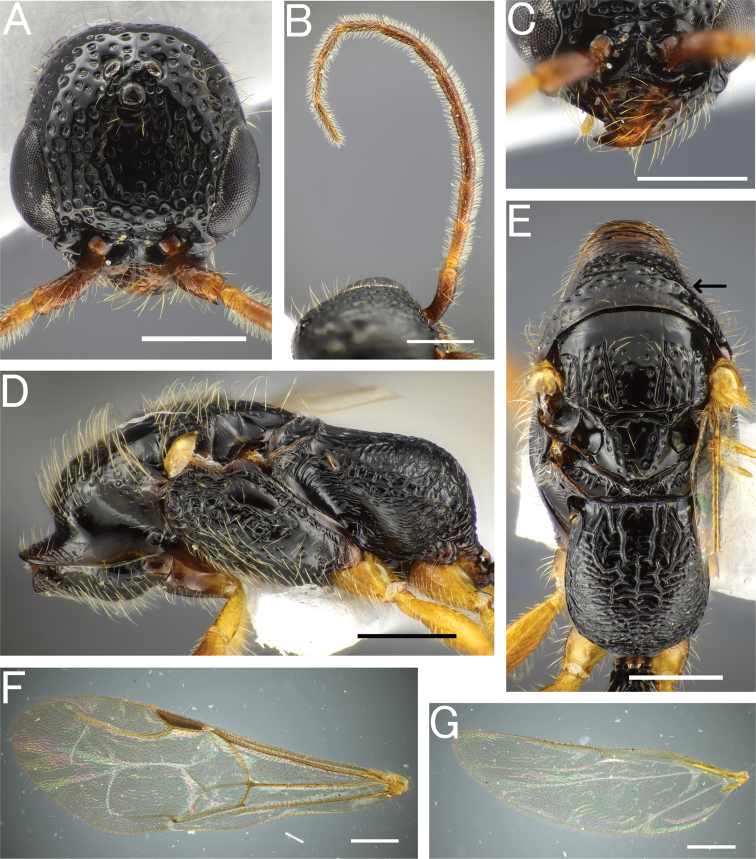
*Pristepyrisseqalu* sp. nov., male, holotype (TP170606_26) **A** head in full-face view **B** antenna (left) **C** mandible **D** mesosoma in lateral view **E** mesosoma in dorsal view; arrow indicating transverse pronotal carina absent **F** fore-wing **G** hindwing. Scale bars: 0.5 mm.

**Figure 5.**
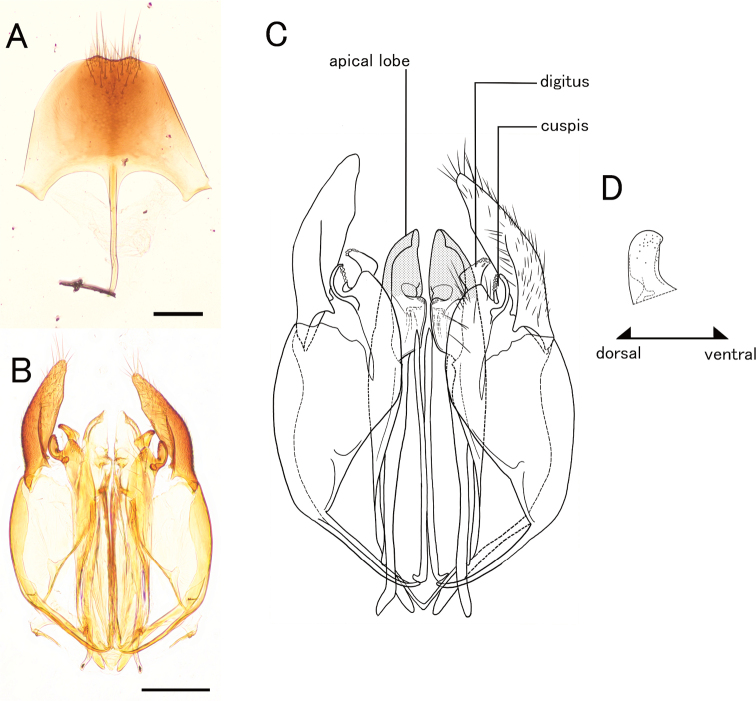
*Pristepyrisseqalu* sp. nov., male genitalia, holotype (TP170606_26) **A** hypopygium **B, C** genitalia in ventral view **D** apical lobe in outer-lateral view. Scale bars: 0.2 mm.

Figs 4 , 5 ; Table 1

###### Male diagnosis.

TL ≈ 5.9–6.0 mm. HL/HW × 100 = 98–105. Frons and vertex with shallow foveolae (ca. 0.03–0.05 mm in diameter), of which intervals are smooth and shining and narrower than diameter of foveolae. Anterior clypeal margin incurved medially. Mandible with five apical teeth. Transverse pronotal carina absent. Cervical pronotal area in lateral view round. LP/WP = 1.02–1.20. Metapostnotal median carina complete posteriorly, but fading in anterior half. Tergum II with weak longitudinal sulcus and weak longitudinal ridge, sternum II without longitudinal median carina. Hypopygium with incurved posterior margin. Apical lobe of aedeagus in lateral view short and lobate, weakly curved ventrad.

###### Female diagnosis.

Unknown.

###### Male description.

***Color*.** Head black; body dark brown; mandible, antenna and legs brown or light brown; fore- and hind-wings subhyaline, with veins brown or light brown.

***Head*.** Head capsule in full-face view evenly round posteriorly, without remarkable posterolateral corner; HL/HW × 100 = 98–105 (98 in holotype). Occipital carina present. Clypeus imbricate, roundly produced anteriad, with median longitudinal carina which not reach anterior clypeal margin; anterior clypeal margin incurved medially. Frons and vertex with deep foveolae (ca. 0.03–0.05 mm in diameter), of which intervals are smooth and shining and narrower than diameter of foveolae. Compound eye large and convex, with sparse thin and relatively short erect setae. POL:AOL = 12:7; OOL:WOT = 4:3; DAO = 0.12 mm. Mandible with five apical teeth; dorsal face faintly imbricate. Antennomere I (excluding basal neck and condylar bulb) 3× as long as maximum width; antennomere I:II:III = 17:3:12 in length; antennomere II 1.4× as long as maximum width, narrowed and bent in basal part; antennomere III–XI each 2.5–4.4× as long as maximum width; antennomere XII 5.2× as long as maximum width, elongate-cylindrical; antennomere XIII (terminal) 6.7× as long as maximum width, with pointed apex.

***Mesosoma*.** Pronotum with pronotal flange extending anteriad beyond anterior margin of propleuron; cervical area in lateral view very steep. Dorsal area of pronotum subtrapezoidal, without distinct transverse pronotal carina (arrow in Fig. 4E), with incurved posterior margin, with deep foveolae, of which intervals are narrower than diameter of foveolae in anterior half, but wider in posterior half; LPD/WPD = 0.39–0.45 (0.39 in holotype). Mesoscutum smooth and shining in anterior 1/3; area along notauli and parapsidal signum foveolate; notaulus distinct in posterior 2/3 of mesoscutum, not reaching posterior margin; parapsidal signum distinct, almost reaching posterior margin of mesoscutum. Mesoscutellum smooth and shining, with sparse and deep foveolae. Mesoscuto-mesoscutellar suture deep and convex anteriad. Mesopleuron elongate; anterior, upper and lower fovea distinct; acropleural area (raised area surrounding anterior, upper and lower fovea) almost smooth and shining. Mesopleural pit absent. Mesodiscrimen concave, with weak median carina. Metasternum with metafurcal pit. Lateral surface of metapectal-propodeal complex obliquely and strongly rugose in marginal area and weakly rugose with intervals shining in central area. Metapectal-propodeal complex in dorsal view with LP/WP = 1.02–1.20 (1.11 in holotype), with lateral margins subparallel, but slightly convex; metapostnotal median carina distinct, almost complete posteriorly, but fading in anterior half; submedian rugae and sublateral margin distinct, but irregularly running; posterior transverse margin indistinct; dorsomedian face sparsely rugoso-scabrous, with intervals smooth and shining; dorsolateral face densely rugoso-scabrous; median portion of propodeal declivity transversely rugoso-scabrous. Forewing with r-m_2_ flexion line (arrows in Fig. 4F), without R_2_ and 2M1_2_ flexion line. Hindwing with five distal hamuli. Claws bifid, with thin and curved apical teeth.

***Metasoma*.** Tergum II with weak longitudinal sulcus and weak longitudinal ridge; sternum II without longitudinal median carina. Hypopygium (subgenital plate) with spiculum much longer than S9ala; apical margin incurved medially; ventral face of apicomedian part with relatively dense setae. Gonostipes glabrous, unfused to harpe. Harpe in ventral view elongate, slightly curved inward, with blunt apex, entirely covered with setae which increase in length toward apex; median basal portion with concavity which accommodates digitus and cuspis. Cuspis lobate and extending laterad, curled, with short, thick, conical setae near apex; subbasal part facing digitus with short and thin hairs. Digitus extending laterad, curled; lateral face with short, thick, conical setae at apex. Apical lobe of aedeagus in lateral view short and lobate, weakly curved ventrad.

###### Female description.

Unknown.

###### Measurements.

***Holotype***: HL 1.16 mm; HW 1.20 mm; EL 0.56 mm; WOT 0.28 mm; POL 0.12 mm; AOL 0.07 mm; OOL 0.40 mm; DAO 0.11 mm; LM 2.25 mm; LPD 0.47 mm; WPD 1.06 mm; LP 0.86 mm; WP 0.74 mm. Paratypes: HL 1.19–1.28 mm; HW 1.19–1.28 mm; EL 0.56–0.61 mm; WOT 0.30–0.32 mm; POL 0.14 mm; AOL 0.10 mm; OOL 0.40–0.42 mm; DAO 0.10 mm; LM 2.25–2.38 mm; LMT 2.68–2.73 mm; LPD 0.48–0.51 mm; WPD 1.10–1.16 mm; LP 0.85–1.00 mm; WP 0.80–0.87 mm; TL 5.9–6.0 mm.

###### Material examined.

***Holotype*.** Mt. Kaoshihfo, Pingtung Country, Taiwan, 22°07'53"N, 120°48'42"E, 483 m alt.; Yoto Komeda leg. (sweeping); 19/V/2017; NSMT. ***Paratypes*.** 2 males (TP170606_11, 13); same data as for holotype; TARI.

###### Etymology.

This species is named after “seqalu”, an aboriginal people who live primarily in Hengchen Township in Taiwan.

###### Taxonomic remarks.

This species is most similar in general appearance to *P.rugulosus* (Terayama et al. 2002) among the named species known from East and Southeast Asia. According to Terayama et al. (2002), posterior transverse margin of metapectal-propodeal complex is distinct in *P.rugulosus*, but indistinct in *P.seqalu* sp. nov.; apical margin of hypopygium is incurved fully in *P.rugulosus*, but only incurved medially in *P.seqalu* sp. nov.; apical lobe of aedeagus in lateral view is relatively small and narrow in *P.rugulosus*, but relatively well-developed, broad and lobate in *P.seqalu* sp. nov.

###### Distribution and habitat.

Southern Taiwan; evergreen broadleaf forest.

##### ﻿ Pristepyris ishigakiensis

Taxon classificationAnimaliaHymenopteraBethylidae

(Yasumatsu, 1955)

98751E60-B4E9-513F-B191-9CC885D22DAD

**Figure 6.**
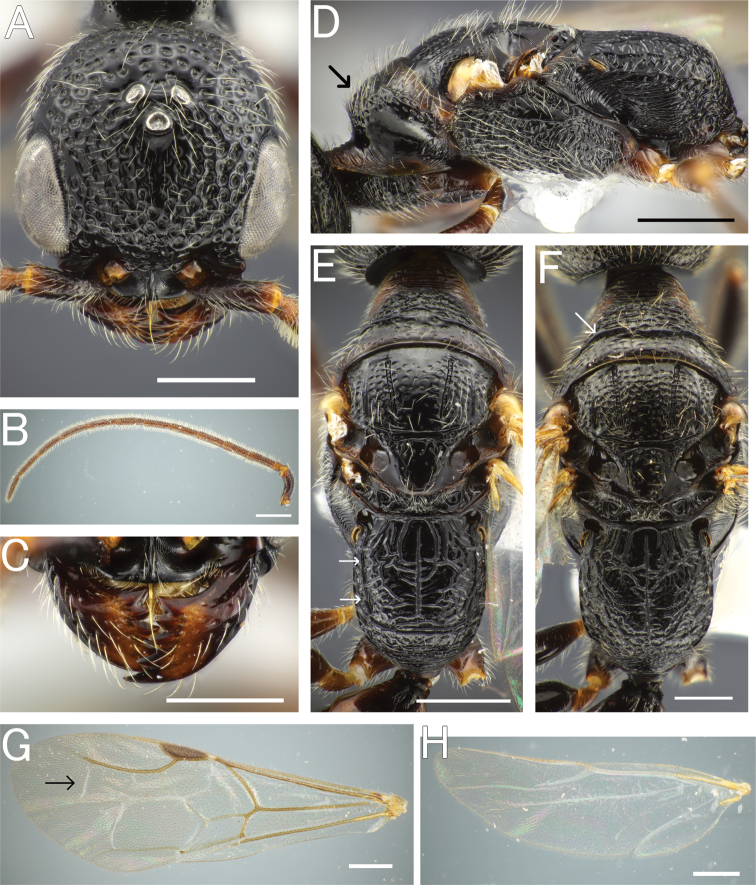
*Pristepyrisishigakiensis*, male, A–E, G, H, JI170808_34, F, TH190717_42 **A** head in full-face view **B** antenna (right) **C** mandible **D** mesosoma in lateral view; arrow indicating an angulate corner present on cervical pronotal area **E** mesosoma in dorsal view; arrows indicating posterior transverse margin extending to spiracle **F** mesosoma in dorsal view; arrow indicating transverse pronotal carina present **G** forewing **H** hindwing. Scale bars: 0.5 mm.

**Figure 7.**
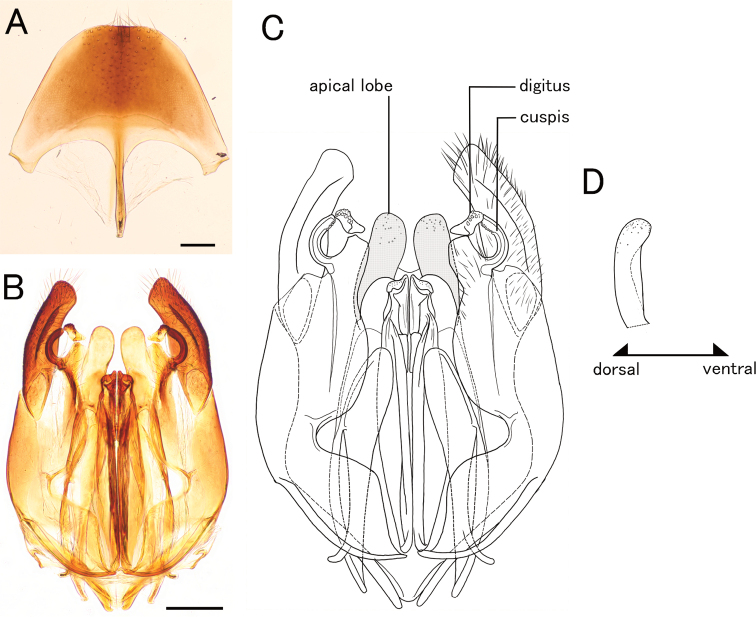
*Pristepyrisishigakiensis*, male genitalia, TH191007_40 **A** hypopygium **B, C** genitalia in ventral view **D** apical lobe in outer-lateral view. Scale bars: 0.2 mm.

Figs 6 , 7 ; Table 1

Pristocera japonica ishigakiensis Yasumatsu, 1955: 245. Holotype (male, KUF), type loc.: Kainan, Ishigaki-jima, Ryukyu Is., Japan. Acrepyrisjaponicaishigakiensis: Terayama, 1996: 595 (genus transfer). Acrepyrisishigakiensis: Terayama, 1999: 103 (raised to species). Pristepyrisishigakiensis: Azevedo et al. 2018: 104 (genus transfer).

###### Male diagnosis.

TL ≈ 6.3–8.0 mm. HL/HW × 100 = 95–100. Frons and vertex with deep foveolae (ca. 0.05–0.06 mm in diameter), of which intervals are smooth and shining and narrower than diameter of foveolae. Anterior clypeal margin nearly straight medially. Mandible with five apical teeth. Transverse pronotal carina present. Cervical pronotal area in lateral view forming an angulate corner. LP/WP = 1.10–1.16. Metapostnotal median carina incomplete posteriorly. Tergum II with longitudinal sulcus and ridge, sternum II with very weak longitudinal median carina or absent. Apical margin of hypopygium straight medially. Apical lobe of aedeagus in lateral view elongate and lobate, directed posteriad, weakly curved ventrad at apex.

###### Female diagnosis.

Unknown.

###### Male redescription.

Full description was given by Yasumatsu (1955) and Terayama (1999). Additional information as below.

***Head*.**HL/HW×100 = 95–100 (98 in holotype). Frons and vertex with deep foveolae (ca. 0.05–0.06 mm in diameter), of which intervals are smooth and usually narrower than diameter of foveolae. Occipital carina present. Clypeus roundly produced anteriad; median clypeal carina moderately distinct, almost reaching anterior clypeal margin; anterior clypeal margin weakly incurved medially (Fig. 4C). Compound eye large and convex, with sparse thin erect setae. Mandible with five teeth.

***Mesosoma*.** Dorsal area of pronotum smooth and shining, with deep foveolae; distinct transverse pronotal carinae present (arrow in Fig. 6F); cervical pronotal area in lateral view forming an angulate corner (arrow in Fig. 6D). Mesopleuron elongate; anterior, upper and lower fovea distinct; acropleural area smooth and shining, with sparse and small foveolae. Mesopleural pit absent. Mesodiscrimen concave, with weak median carina. Metasternum with metafurcal pit. Lateral face of metapectal-propodeal complex irregularly rugose entirely. Metapectal-propodeal complex in dorsal view with LP/WP = 1.10–1.16, with lateral margins subparallel and slightly convex; metapostnotal median carina distinct, but incomplete posteriorly; submedian rugae irregularly running and incomplete posteriorly; sublateral margin incomplete posteriorly; posterior transverse margin indistinct or distinctly extending to spiracle (Fig. 6E); dorsomedian and dorsolateral faces weakly rugoso-scabrous; median portion of propodeal declivity transversely rugoso-scabrous. Forewing with r-m_2_ flexion line (arrows in Fig. 6G), without R_2_ and 2M1_2_ flexion line. Hindwing with five distal hamuli. Tarsal claws bifid, with thin and curved apical teeth.

***Metasoma*.** Tergum II with longitudinal sulcus and ridge; sternum II with very weak longitudinal median carina or absent. Hypopygium (subgenital plate) with spiculum much longer than S9ala; apical margin straight medially; ventral face of apicomedian part with relatively dense setae. Gonostipes glabrous, unfused to harpe. Harpe in ventral view elongated, slightly curved inward, with blunt apex, entirely covered with setae which increase in length toward apex; median basal portion with concavity which accommodates digitus and cuspis. Cuspis lobate and extending laterad, curled, with short, thick, conical setae near apex; subbasal part facing digitus with short and thin hairs. Digitus extending laterad, curled; lateral face with short, thick, conical setae at apex. Apical lobe of aedeagus in lateral view elongate and lobate, directed posteriad, weakly curved ventrad at apex.

###### Female description.

Unknown.

###### Material examined.

**Japan: Ishigaki**–**jima.** 3 males (JI170808_30, 33, 34); Mt. Omoto, 24°26'31"N, 124°05'56"E, 93 m alt.; Hauchuan Liao leg. (sweeping); 12/VIII/2017. 1 male (JI170808_36); Mt. Yarabu, 24°26'22"N, 124°05'32"E, 154 m alt.; Hauchuan Liao leg. (sweeping); 13/VIII/2017. **Taiwan: N. Taiwan.** 1 male (TT91007_06); Dagoushi Park, Taipei City, 25°05'20"N, 121°35'38"E, 81 m alt.; Hauchuan Liao leg. (sweeping); 9/X/2019. **E. Taiwan.** 5 males (TH191007_27, 37, 40, 41, 42); TsoTsang Trail, Hualien County, 24°00'53"N, 121°34'18"E, 266 m alt.; Hauchuan Liao leg. (sweeping); 24/X/2019. **S. Taiwan.** 1 male (TP171019_10); Baoli Experimental Forest, Pingtung County, 24°04'15"N, 120°45'51"E, 79 m alt.; Hauchuan Liao leg. (sweeping); 22/X/2017.

###### Taxonomic remarks.

In our collection, a specimen from Ishigaki–jima Island has the posterior transverse margin of metapectal-propodeal complex that is distinct and extends to spiracle distinctly (Fig. 6E) and the other specimens have the margin that is indistinct as in the original description (Fig. 6F). However, there are no remarkable differences between the two forms in male genital morphology and in both the 28S and COI sequences. This fact suggests the conspecificity of the two forms (these are likely geographic variations of a single species).

###### Distribution and habitat.

Southern Ryukyus (Terayama 2006), from the north to south of Taiwan (new to Taiwan); evergreen broadleaf forest.

##### ﻿ Pristepyris mieae

Taxon classificationAnimaliaHymenopteraBethylidae

(Terayama, 1995)

1B042A90-3B57-5036-921E-C618103D4605

Fig. 8

Acrepyris mieae Terayama, 1995: 142, figs 10. Holotype (female, NIAES), type loc.: Fenchifu Chiayi Hsien, Taiwan. Pristepyrismieae: Azevedo et al. 2018: 104 (genus transfer).

###### Male diagnosis.

Unknown.

###### Female diagnosis.

TL ≈ 6.3 mm. Frons and vertex with deep foveolae (ca. 0.03 mm in diameter), of which intervals are imbricate; intervals in vertex wider than diameter of foveolae; intervals in lateral and submedian part of frons narrower than diameter of foveolae; the area along mesal line without foveolae. Median portion of clypeus roundly and relatively strongly produced anteriad; apical clypeal margin deeply incurved medially. Compound eye less developed. Mandible with four teeth. Dorsal face of pronotum, mesoscutellum, mesopleuron and dorsal and lateral faces of metapectal-propodeal complex imbricate, with dense foveolae. Transverse pronotal carina absent.

###### Female redescription.

Full description was given by Terayama (1995). Additional information as below.

***Head*.**HL/HW × 100 = 131. Frons and vertex with deep foveolae (ca. 0.03 mm in diameter), of which intervals are imbricate; intervals in vertex wider than diameter of foveolae; intervals in lateral and submedian part of frons narrower than diameter of foveolae; the area along mesal line without foveolae. Occipital carina present. Median portion of clypeus roundly and relatively strongly produced anteriad; apical clypeal margin deeply incurved medially.

***Mesosoma*.** Transverse pronotal carina absent. Dorsal area of pronotum imbricate with dense foveolae. Mesoscutum overlaid by posteromedian portion of pronotum. Mesoscutellum trapezoidal, 0.67× as long as maximum width, weakly imbricate with dense foveolae. Mesopleuron imbricate, with sparse foveolae; anterior, upper and lower fovea absent; mesopleural pit absent. Lateral face of metapectal-propodeal complex imbricate entirely. Metapectal-propodeal complex in dorsal view weakly constricted behind propodeal spiracles and then widened again posteriad, without any distinct carinae which subdivide dorsal face; LP/WP = 2.5; dorsomedian face weakly imbricate, with sparse foveolae; median portion of propodeal declivity weakly and transversely rugoso-scabrous, with sparse foveolae.

###### Taxonomic remarks.

This species is morphologically most similar to the female of *P.zhejiangensis*. However, the female specimens of the genus *Pristepyris* have been rarely recorded and female-based species discrimination is hard to be conducted because of poor diagnostic characters in the females. We tentatively treated *P.mieae* as an independent species until additional specimens are available for molecular analyses.

**Figure 8.**
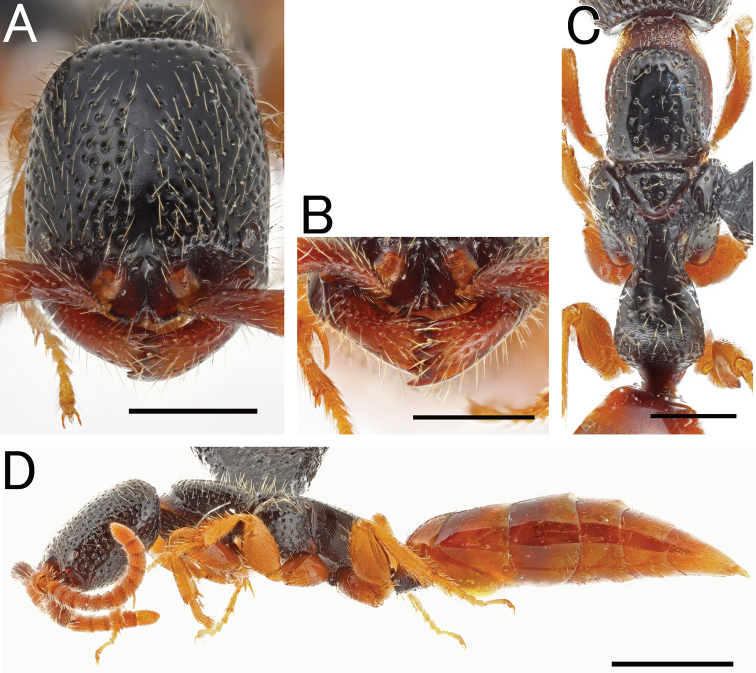
*Pristepyrismieae*, female, holotype **A** head in full-face view **B** mandible **C** mesosoma in dorsal view **D** mesosoma in lateral view. Scale bars: 0.5 mm.

##### ﻿ Pristepyris ryukyuensis

Taxon classificationAnimaliaHymenopteraBethylidae

(Terayama, 1999)

0C22F5D6-7819-5FEF-B6E1-799B85FDC529

Fig. 9

Acrepyris ryukyuensis Terayama, 1999: 702, figs 1, 2. Holotype (male, NIAES), type loc.: Shimoji, Miyako-jima, Okinawa, Japan. Pristepyrisryukyuensis: Azevedo et al. 2018: 104 (genus transfer).

###### Male diagnosis.

HL/HW × 100 = 105. Frons and vertex with deep foveolae (ca. 0.05–0.06 mm in diameter), of which intervals are smooth and shining and narrower than diameter of foveolate. Anterior clypeal margin nearly straight medially. Mandible with five apical teeth. Transverse pronotal carina present. Cervical pronotal area in lateral view strongly and roundly produced. LP/WP = 1.09. Metapostnotal median carina incomplete posteriorly.

###### Female diagnosis.

Unknown.

###### Male redescription.

Full description was given by Terayama (1999). Additional information as below.

***Head*.**HL/HW×100 = 105. Frons and vertex with deep foveolae (ca. 0.05–0.06 mm in diameter), of which intervals are smooth and shining and narrower than diameter of foveolate. Occipital carina present. Median portion of clypeus roundly produced anteriad; median clypeal carina moderately distinct, almost reaching anterior margin; anterior clypeal margin nearly straight medially. Compound eye large and convex, with sparse thin erect setae. Mandible with five teeth.

**Figure 9.**
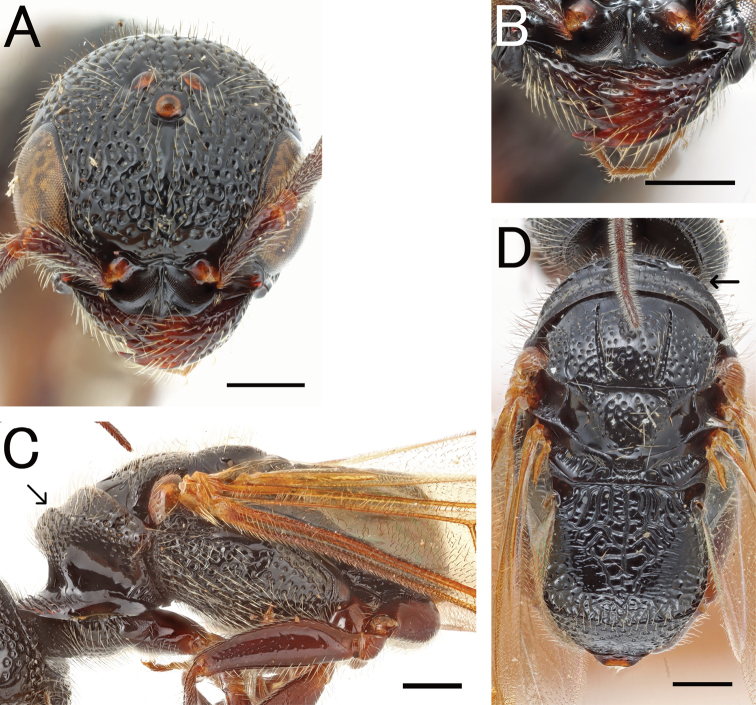
*Pristepyrisryukyuensis*, male, holotype **A** head in full-face view **B** mandible **C** mesosoma in lateral view; arrow indicating an angulate corner present on cervical pronotal area **D** mesosoma in dorsal view; arrow indicating transverse pronotal carina present. Scale bars: 0.5 mm.

***Mesosoma*.** Dorsal area of pronotum smooth and shining, with deep foveolae; distinct transverse carinae present (arrow in Fig. 9D); cervical pronotal area in lateral view strongly and roundly produced (arrow in Fig. 9C). Mesopleuron elongate; anterior, upper and lower fovea distinct; acropleural area smooth and shining. Mesopleural pit absent. Metapectal-propodeal complex in dorsal view with LP/WP = 1.09, with lateral margins subparallel and slightly convex; metapostnotal median carina distinct, but incomplete posteriorly; submedian rugae irregularly running; sublateral margin distinct, incomplete posteriorly; posterior transverse margin distinct; dorsomedian and dorsolateral faces weakly rugoso-scabrous; median portion of propodeal declivity weakly rugoso-scabrous.

***Metasoma*.** Missing.

###### Female description.

Unknown.

###### Taxonomic remarks.

This species is most similar to *Pristepyriszhejiangensis.* The two species share two remarkable features: mandible is five-toothed; cervical pronotal area in lateral view is strongly and roundly produced (arrow in Fig. 9C). However, the type material (holotype only) of *P.ryukyuensis* lacks the mesosoma and no metasomal and genital morphology is given in the original description. Therefore, it is impossible to conclude whether the two morphospecies are conspecific or not. *Pristepyrisryukyuensis* is tentatively treated as an independent species of which the identity will be discussed, based on the further intensive sampling in the whole of the potential distributional range (the Ryukyus, Taiwan and the eastern coastal region of mainland China). Furthermore, the *P.ryukyuensis*-like and *P.zhejiangensis*-like specimens newly obtained from the Ryukyus and Taiwan were treated as *P.zhejiangensis*, based on the reliable male genital morphology.

##### ﻿ Pristepyris tainanensis

Taxon classificationAnimaliaHymenopteraBethylidae

(Terayama, 1995)

F36E9931-E31E-5E84-B88E-085829725487

Fig. 10

Acrepyris tainanensis Terayama, 1995: 143, figs 11–14. Holotype (male, HUS), type loc.: Raisha, Taiwan; paratype (male, HUS), type loc.: Kanshirei; paratype (male, NIAES), type loc.: Kuanzuling, Tainan Hsien. Pristepyristainanensis: Azevedo et al. 2018: 104 (genus transfer).

###### Male diagnosis.

TL ≈ 8.6 mm. HL/HW × 100 = 103. Frons and vertex with shallow foveolae (ca. 0.05–0.06 mm in diameter), of which intervals are smooth and shining and narrower than diameter of foveolae. Anterior clypeal margin nearly straight medially. Mandible with five apical teeth. Transverse pronotal carina present. Cervical pronotal area in lateral view forming an angulate corner. LP/WP = 0.96. Metapostnotal median carina not complete posteriorly.

###### Female diagnosis.

Unknown.

###### Male redescription.

Full description was given by Terayama (1995). Additional information as below.

***Head*.** Frons and vertex with deep foveolae (ca. 0.05–0.06 mm in diameter), of which intervals are smooth and shining; intervals in vertex and frons usually narrower than diameter of foveolae. Occipital carina present. Median portion of clypeus roundly produced anteriad; median clypeal carina moderately distinct, almost reaching anterior margin; anterior clypeal margin truncate, nearly straight medially. Compound eye large and convex. Mandible with five teeth.

**Figure 10.**
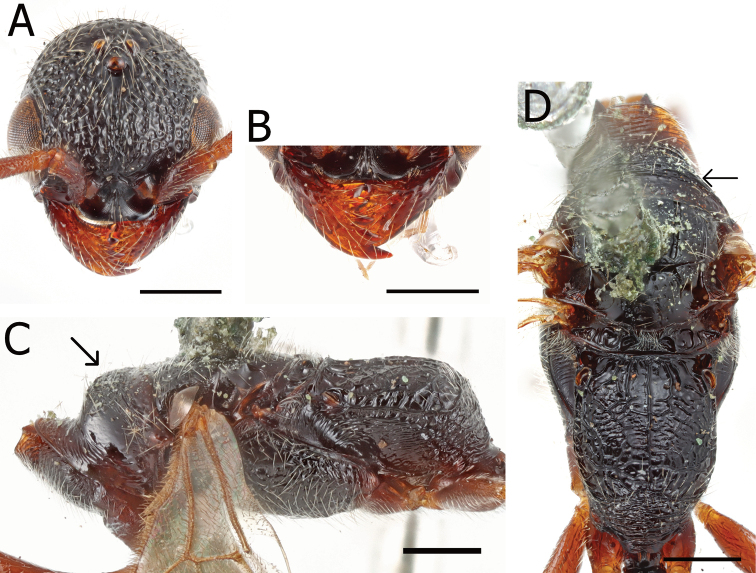
*Pristepyristainanensis*, male, paratype **A** head in full-face view **B** mandible **C** mesosoma in lateral view (mirror-reversed); arrow indicating an angulate corner present on cervical pronotal area **D** mesosoma in dorsal view; arrow indicating transverse pronotal carinae present. Scale bars: 0.5 mm.

***Mesosoma*.** Dorsal area of pronotum smooth and shining, with deep foveolae; distinct transverse carina(e) present (arrow in Fig. 10D); cervical pronotal area in lateral view forming an angulate corner (arrow in Fig. 10C). Mesopleuron elongate; anterior, upper and lower fovea distinct; acropleural area smooth and shining. Mesopleural pit absent. Lateral face of metapectal-propodeal complex irregularly rugose. Metapectal-propodeal complex in dorsal view with LP/WP = 0.96, with lateral margins subparallel and slightly convex; metapostnotal median carina distinct, but incomplete posteriorly; submedian rugae irregularly running and incomplete posteriorly; sublateral margin incomplete posteriorly; posterior transverse margin weak; dorsomedian and dorsolateral faces weakly rugoso-scabrous; median portion of propodeal declivity transversely rugoso-scabrous.

###### Female description.

Unknown.

###### Taxonomic remarks.

This species is most similar to *Pristepyrisishigakiensis.* The two species share two remarkable features: mandible is five-toothed; cervical pronotal area in lateral view forming an angulate corner (arrow in Figs 6D, 10C). However, no metasomal and genital morphology is given in the original description of *P.tainanesis* and the present authors had no opportunity to dissect and examine the male genitalia of the type material. Therefore, it is impossible to conclude whether the two morphospecies are conspecific or not and *P.tainanensis* is tentatively treated as an independent species. The identity will be discussed when the “topotypes” of *P.tainanesis* become available in the future.

###### Distribution and habitat.

Southern Taiwan.

##### ﻿ Pristepyris zhejiangensis

Taxon classificationAnimaliaHymenopteraBethylidae

(Terayama, Xu & He, 2002)

960BB0F4-DC9A-57DE-A6A8-5910EC3582B1

**Figure 11.**
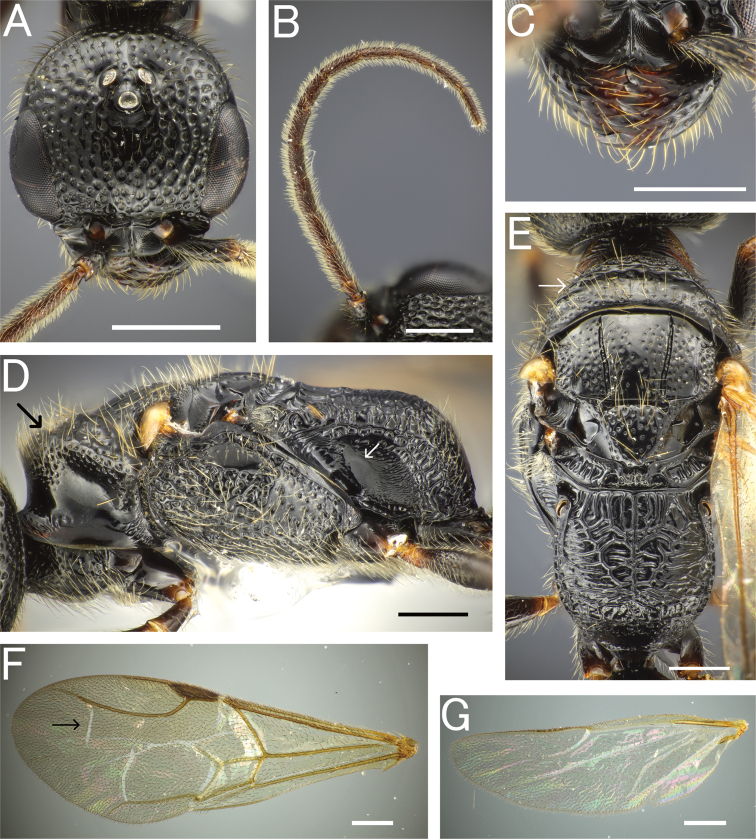
*Pristepyriszhejiangensis*, male **A–C, F, G** TNT180504_01 **D, E** JM190717_33 **A** head in full-face view **B** antenna (left) **C** mandible **D** mesosoma in lateral view; arrow indicating an angulate corner present on cervical pronotal area **E** mesosoma in dorsal view; arrow indicating transverse pronotal carina present **F** forewing **G** hindwing. Scale bars: 0.5 mm.

**Figure 12.**
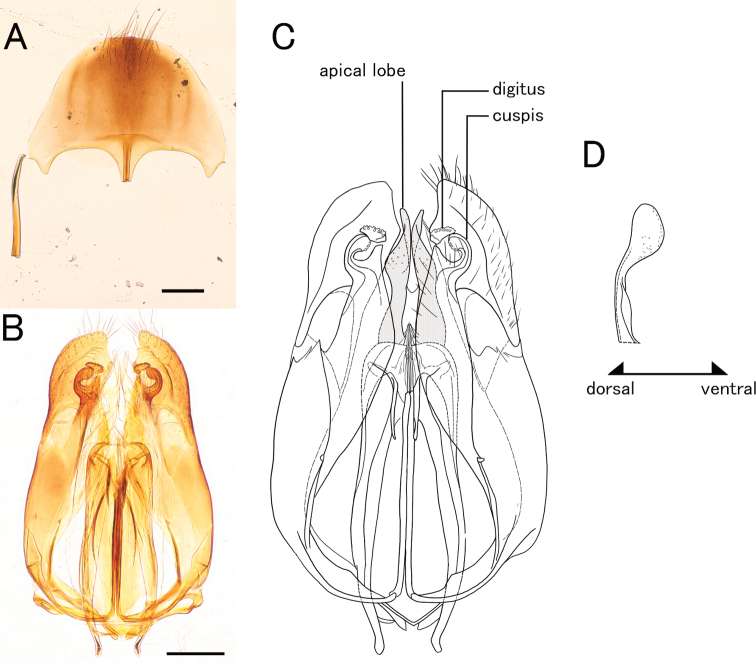
*Pristepyriszhejiangensis*, male genitalia, TNT180504_01 **A** hypopygium **B, C** genitalia in ventral view **D** apical lobe in outer-lateral view. Scale bars: 0.2 mm.

**Figure 13.**
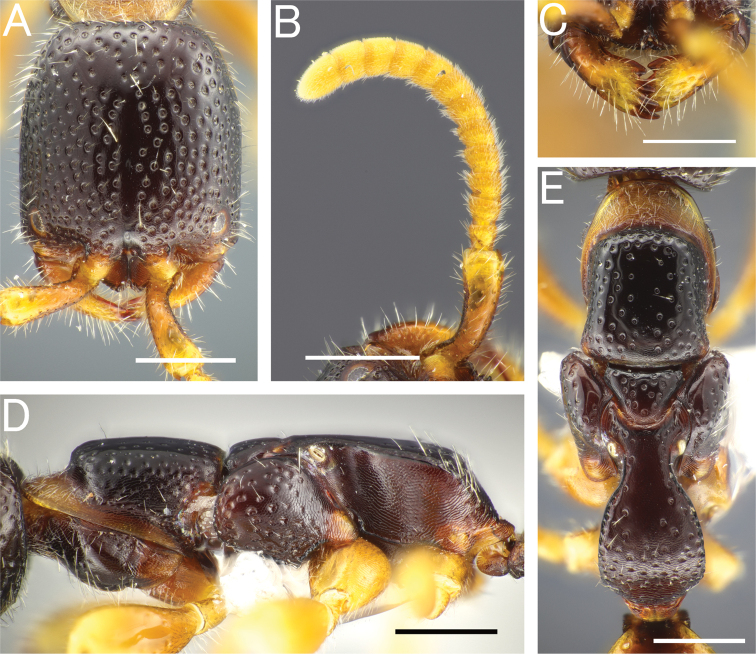
*Pristepyriszhejiangensis*, female, TN170427_01 **A** head in full-face view **B** antenna (left) **C** mandible **D** mesosoma in lateral view **E** mesosoma in dorsal view. Scale bars: 0.5 mm.

Figs 11 , 12 , 13 ; Table 1

Acrepyris zhejiangensis Terayama et al. 2002: 83, figs 9–16. Holotype, type loc.: Deqing, Zhejiang, China. Pristepyriszhejiangensis: Azevedo et al. 2018: 104 (genus transfer).

###### Male diagnosis.

TL ≈ 6.1–9.3 mm. HL/HW × 100 = 88–103. Frons and vertex with deep foveolae (ca. 0.05–0.06 mm in diameter), of which intervals are smooth and shining and narrower than diameter of foveolate. Anterior clypeal margin nearly straight medially. Mandible with five apical teeth. Transverse pronotal carina present. Cervical pronotal area in lateral view round. LP/WP = 0.97–1.04. Metapostnotal median carina incomplete posteriorly. Tergum II with longitudinal sulcus and ridge, sternum II with longitudinal median carina. Apical margin of hypopygium straight medially. Apical lobe of aedeagus in lateral view elongate and spatulate, with broadened and rounded apex, in ventral view somewhat winding.

###### Female diagnosis.

TL ≈ 6.5 mm. HL/HW×100 = 118–126. Frons and vertex with deep foveolae (ca. 0.03–0.04 mm in diameter), of which intervals are imbricate; intervals in vertex wider than diameter of foveolae; intervals in lateral and submedian part of frons as narrow as or narrower than diameter of foveolae; the area along mesal line without foveolae. Median portion of clypeus roundly and relatively strongly produced anteriad; apical clypeal margin deeply incurved medially. Compound eye less developed. Mandible with four teeth. Transverse pronotal carina absent. Dorsal face of pronotum, mesoscutellum, mesopleuron and dorsal and lateral faces of metapectal-propodeal complex imbricate. Mesosoma excluding dorsal and lateral faces of metapectal-propodeal complex with dense foveolae. Tarsal claws with thin and curved tooth. Tergum II with weak longitudinal ridge, without longitudinal sulcus. Sternum II without longitudinal median carina.

###### Male redescription.

Full description was given by Terayama et al. (2002). Additional information as below.

***Head*.**HL/HW×100 = 88–103 (88 in holotype). Frons and vertex with deep foveolae (ca. 0.05–0.06 mm in diameter), of which intervals are smooth and shining and narrower than diameter of foveolate. Occipital carina present. Median portion of clypeus roundly produced anteriad; median clypeal carina moderately distinct, almost reaching anterior margin; anterior clypeal margin nearly straight medially. Compound eye large and convex, with sparse thin erect setae. Mandible with five teeth.

***Mesosoma*.** Dorsal area of pronotum smooth and shining, with deep foveolae, with distinct transverse pronotal carinae (arrow in Fig. 8E). Cervical pronotal area in lateral view round. Mesopleuron elongate; anterior, upper and lower fovea distinct; acropleural area smooth and shining. Mesopleural pit absent. Mesodiscrimen concave, with weak median carina. Metasternum with metafurcal pit. Lateral face of metapectal-propodeal complex smooth and shining anteriorly, irregularly rugose on posterior half of central area. Metapectal-propodeal complex in dorsal view with LP/WP = 0.97–1.04, with lateral margins subparallel and slightly convex; metapostnotal median carina distinct, but incomplete posteriorly; submedian rugae irregularly running; sublateral margin distinct, incomplete posteriorly; posterior transverse margin distinct; dorsomedian and dorsolateral faces weakly rugoso-scabrous; median portion of propodeal declivity transversely rugoso-scabrous. Forewing with r-m_2_ flexion line (arrows in Fig. 8F), without R_2_ and 2M1_2_ flexion line. Hindwing with five distal hamuli. Tarsal claws bifid, with thin and curved apical teeth.

***Metasoma*.** Tergum II with longitudinal sulcus and ridge; sternum II with longitudinal median carina. Hypopygium with spiculum much longer than S9ala (spiculum broken in Fig. 9A); apical margin straight medially; ventral face of apicomedian part with relatively dense setae. Gonostipes glabrous, unfused to harpe. Harpe in ventral view widely elongated, slightly curved inward, with blunt apex, entirely covered with setae which increase in length toward apex; median basal portion with concavity which accommodates digitus and cuspis. Cuspis lobate and extending laterad, curled, with short, thick, conical setae at apex; subbasal part facing digitus with short and thin setea. Digitus extending laterad, curled; lateral face with short, thick, conical setae near apex. Apical lobe of aedeagus in lateral view elongate and spatulate, with broadened and rounded apex, in ventral view somewhat winding.

###### Female description.

Female of this species was newly-recognised by molecular phylogenetic analyses in the present study.

***Color*.** Body mostly dark brown; mandible, antenna, anterior flange of pronotum and legs brown or light brown.

***Head*.** Head capsule with posterior margin slightly incurved, with posterolateral corner round; HL/HW × 100 = 118–126. Occipital carina present. Frons and vertex with deep foveolae (ca. 0.03–0.04 mm in diameter), of which intervals are imbricate; intervals in vertex wider than diameter of foveolae; intervals in lateral and submedian part of frons as narrow as or narrower than diameter of foveolae; the area along mesal line without foveolae. Median portion of clypeus roundly and relatively strongly produced anteriad, imbricate; median longitudinal carina not reaching anterior clypeal margin; anterior clypeal margin deeply incurved medially. Compound eye less developed. Mandible with four teeth; basalmost tooth relatively shorter than other ones. Antennomere I (excluding basal condylar bulb) 2.7× as long as maximum width; antennomere I:II:III = 5:1:1 in length; antennomere II 0.9× as long as maximum width, narrowed and bent in basal part; antennomere III–XII each 0.76–0.85× as long as maximum width, elongate-cylindrical; antennomere XIII (terminal) 1.3× as long as maximum width, with round apex. Tarsal claws with thin and curved tooth.

***Mesosoma*.** Pronotum with anterior flange extending anteriad beyond anterior margin of propleuron; transverse pronotal carina absent; cervical pronotal area in lateral view round, with a steep anterior face; dorsal area subtrapezoidal, with almost straight posterior margin, with deep foveolae of which intervals are wider than diameter of foveolae and weakly imbricate; LPD/WPD = 1.00–1.07. Mesoscutum overlain by posteromedian portion of pronotum. Mesoscutellum trapezoidal, 0.63–0.64× as long as maximum width, weakly imbricate, with sparse and deep foveolae. Mesopleuron largely imbricate excluding smooth anterodorsal part, with sparse and deep foveolae; anterior, upper and lower fovea absent; mesopleural pit absent. Mesodiscrimen with weak median carina. Metasternum with metafurcal pit. Lateral face of metapectal-propodeal complex imbricates entirely. Metapectal-propodeal complex in dorsal view weakly constricted behind propodeal spiracles and then widened again posteriad, without any distinct carinae which subdivide dorsal face; LP/WP = 2.28–2.42; dorsomedian face weakly imbricate; median portion of propodeal declivity weakly and transversely rugoso-scabrous, with sparse foveolae.

***Metasoma*.** Tergum II with weak longitudinal ridge, without longitudinal sulcus; sternum II without longitudinal median carina.

###### Material examined.

**Japan: Irabu**–**jima.** 15 males (JM190717_31–45); Makiyama Park, 24°48'57"N, 125°13'00"E, 93 m alt.; HauChuan Liao leg. (sweeping); 23/VII/2019. 1 female (JM190717_28); Makiyama Park, 24°48'57"N, 125°13'00"E, 93 m alt.; HauChuan Liao leg. (sweeping); 23/VII/2019. **Iriomote**–**jima** 1 male (JIR190717_47); Tropical Biosphere Research Center, 24°23'48"N, 123°48'11"E, 33 m alt. HauChuan Liao leg. (sweeping). **Taiwan: N. Taiwan.** 2 males (TNT171019_04, TNT180504_01); Mt. Dadao Wurai, New Taipei City, 24°51'09"N, 121°33'27"E, 548 m alt.; Hauchuan Liao leg. (sweeping); 26/X/2017, 4/V/2018. **C. Taiwan.** 1 male (TN190315_24); Sungpolun Trail, Nantou County, 23°52'06"N, 120°55'44"E, 789 m alt.; HauChuan Liao leg. (sweeping); 20/III/2019. 1 female (TN170427_01); Huisun Experimental Forest, Nantou County. Po-Cheng Hsu leg.; 27/IV/2017.

###### Taxonomic remarks.

This species is most similar in general appearance to *P.ryukyuensis* among the named species known from East and Southeast Asia (for details, see under Taxonomic remarks of “*P.ryukyuensis*”).

###### Distribution and habitat.

Eastern China (Zhejiang), southern Ryukyu, northern and central Taiwan (new to Taiwan); evergreen broadleaf forests.

### ﻿Key to Taiwanese and Ryukyuan species of the genus *Pristepyris*, based on male morphology

As mentioned above, the present study was unable to provide any evidence which supports or rejects the discrimination between *P.ryukyuensis* and *P.tainanensis* and between *P.ryukyuensis* and *P.zhejiangensis*. *Pristepyrisishigakiensis* was also unable to be discriminated from *P.tainanensis* morphologically. Therefore, these morphological forms are treated as “*P.zhejiangensis* species complex” and “*P.ishigakiensis* species complex”, respectively, in the following key and are likely *P.ryukyuensis* or *P.tainanensis*. Female-based species, *P.mieae*, of which the male is unknown, is also omitted from the following key.

**Table:** 

1	Transverse pronotal carina absent; apical lobe of aedeagus in lateral view short and lobate (Fig. 5C)	***P.seqalu* sp. nov.**
–	Distinct transverse pronotal carinae present; apical lobe of aedeagus in lateral view elongate and lobate (Fig. 7C) or elongate and spatulate (Fig. 12C)	**2**
2	Cervical pronotal area in lateral view strongly and roundly produced (black arrow in Fig. 11D); apical lobe of aedeagus in ventral view winding (Fig. 12C), in lateral view elongate and spatulate (Fig. 12D)	***P.zhejiangensis* species complex**
–	Cervical pronotal area in lateral view forming an angulate corner (arrow in Fig. 6D), but not strongly and roundly produced; apical lobe of aedeagus in ventral view straight, not winding (Fig. 7C), in lateral view elongate and lobate (Fig. 7D)	***P.ishigakiensis* species complex**

#### *Eleganesia* Alencar & Azevedo, 2018

##### ﻿ Eleganesia minuta

Taxon classificationAnimaliaHymenopteraBethylidae

(Yasumatsu, 1955) comb. nov.

5A3C338E-9BA7-5C1A-BDE1-6E935BC7927C

**Figure 14.**
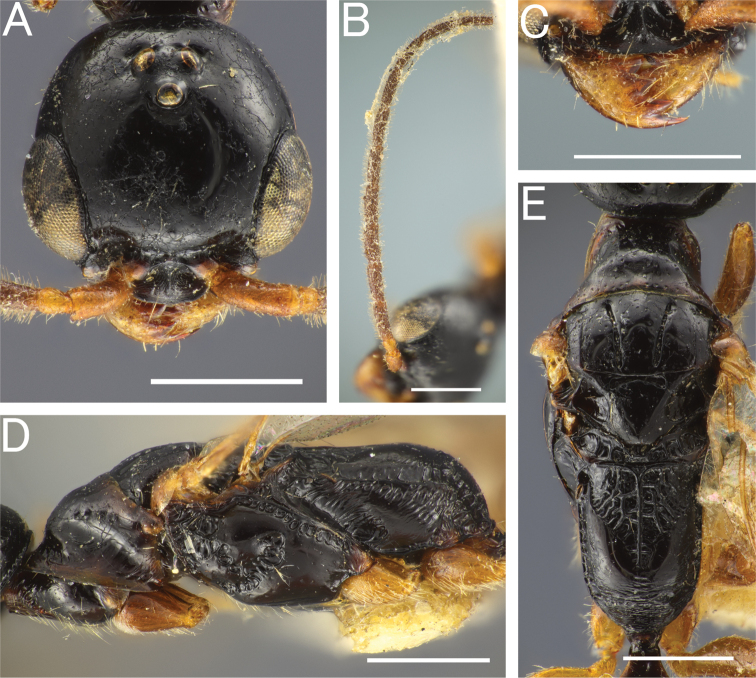
*Eleganesiaminuta* comb. nov., male, holotype **A** head in full-face view **B** antenna (right) **C** mandible **D** mesosoma in lateral view **E** mesosoma in dorsal view **F** Forewing **G** hindwing. Scale bars: 0.5 mm.

**Figure 15.**
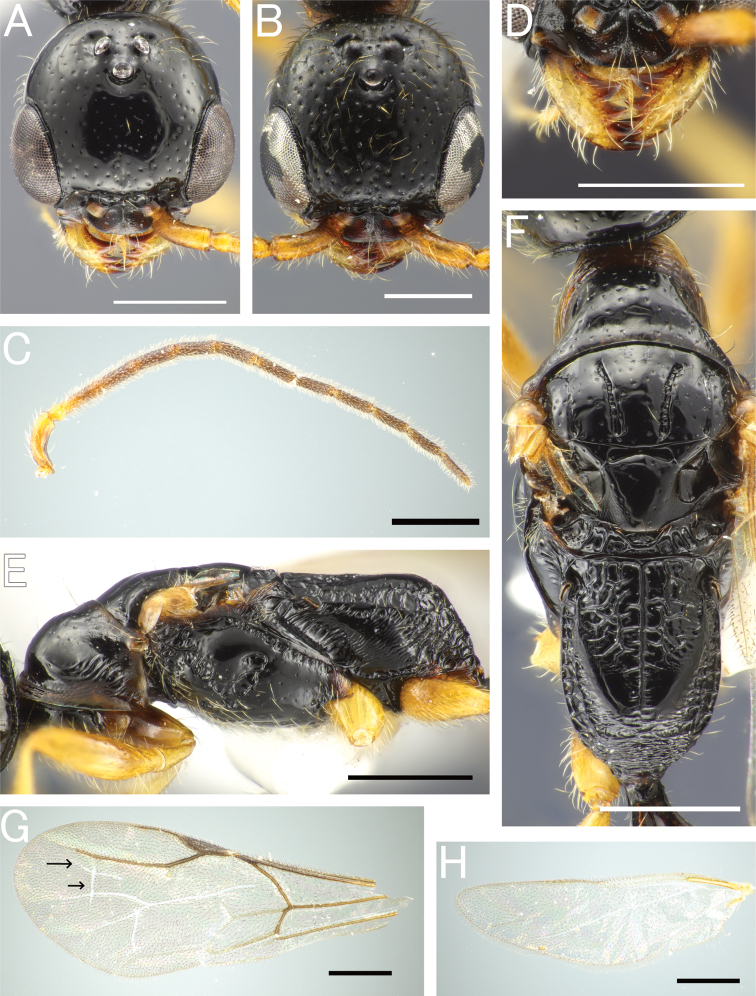
*Eleganesiaminuta* comb. nov., male **A, C–H** JO190717_13 **B** JIR190717_54 **A, B** head in full-face view **C** antenna (left) **D** mandible **E** mesosoma in lateral view **F** mesosoma in dorsal view **G** forewing **H** hindwing. Scale bars: 0.5 mm.

**Figure 16.**
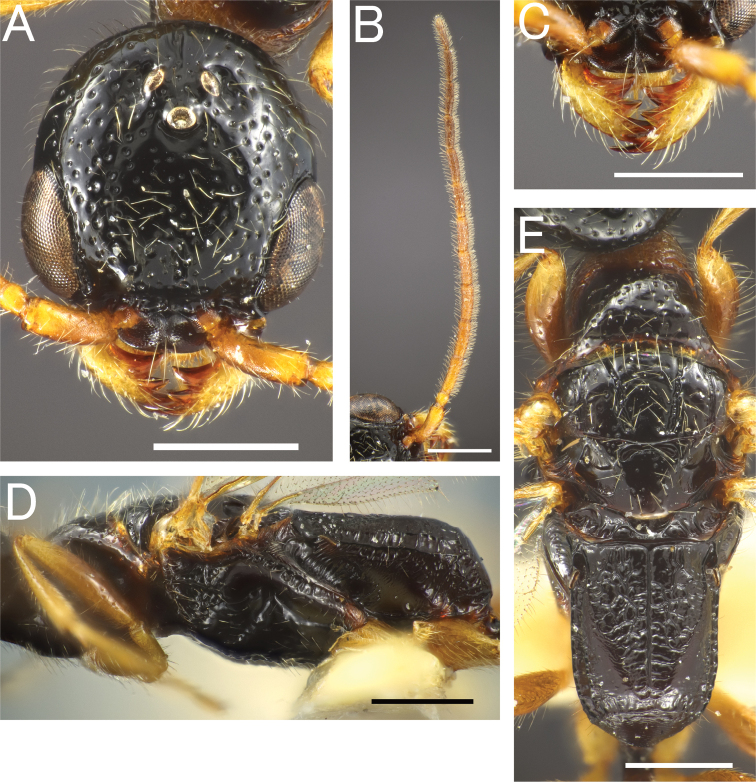
“*Pristepyristakasago*”, male, holotype **A** head in full-face view **B** antenna (right) **C** mandible **D** mesosoma in lateral view **E** mesosoma in dorsal view **F** forewing **G** hindwing. Scale bars: 0.5 mm.

**Figure 17.**
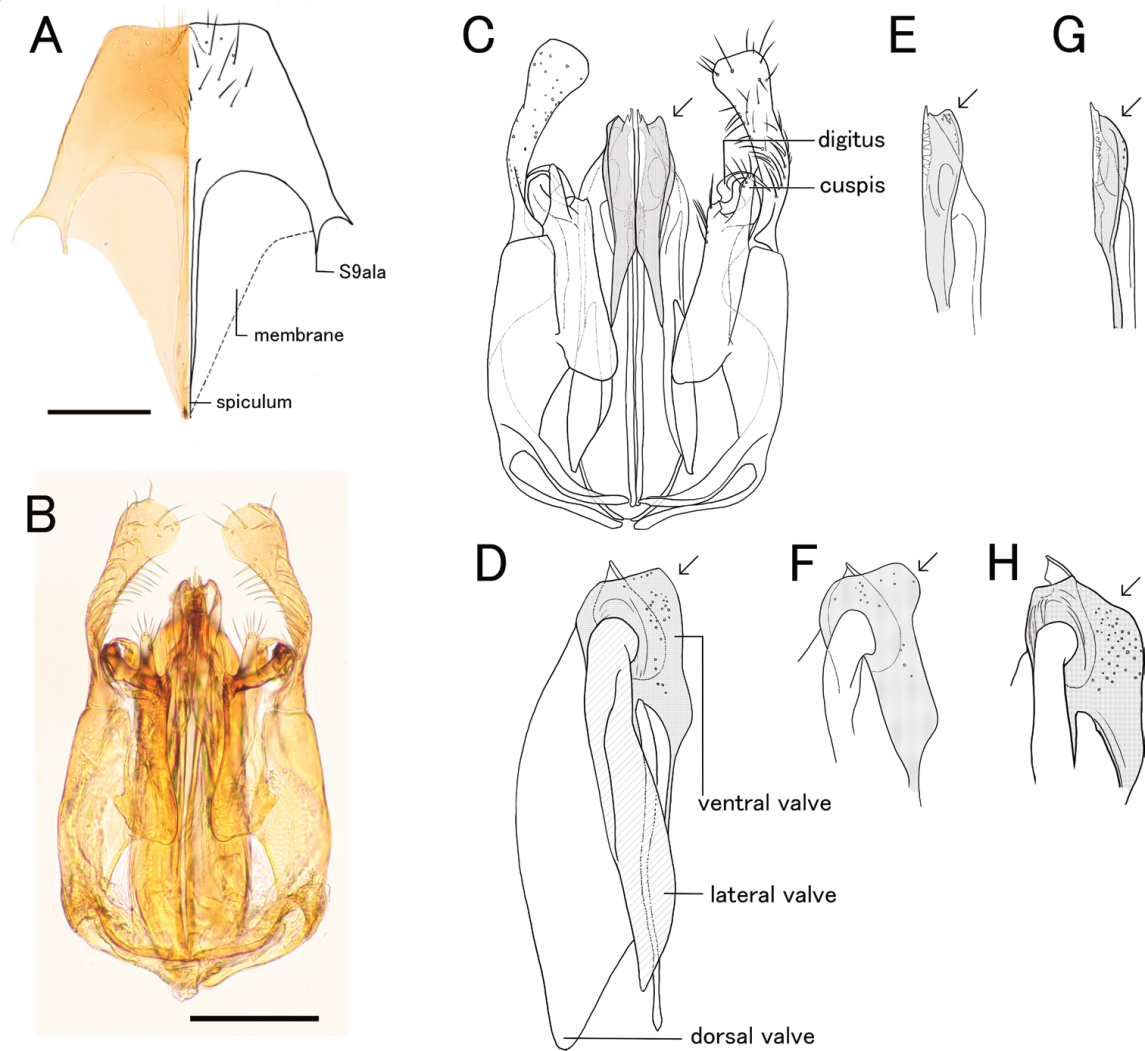
*Eleganesiaminuta* comb. nov., male genitalia **A, C** (JO190717_13) from Okinawa-Hontou Island **B** (holotype) **D** (JA170808_13) from Amami-Oshima Island **E** (JI170808_31) **F** (JI170808_35) from Ishigaki–jima Island **G** (TNT180706_01) **H** (TNT180706_06) from Taiwan **A** hypopygium **B, C, E, G** genitalia (and aedeagus) in ventral view **D, F, H** aedeagus in outer-lateral view; arrows show morphological variation in ventral valve of aedeagus. Scale bars: 0.2 mm.

**Figure 18.**
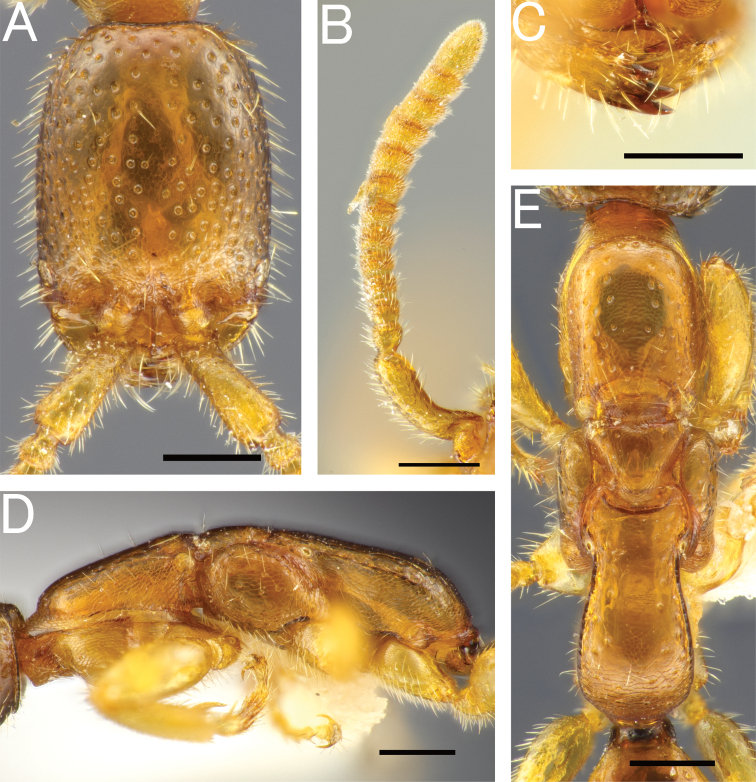
*Eleganesiaminuta* comb. nov., female **A** head in full-face view **B** antenna (left) **C** mandible **D** mesosoma in lateral view **E** mesosoma in dorsal view. Scale bars: 0.2 mm.

Figs 14 , 15 , 16 , 17 , 18 ; Table 1

Pristocera minuta Yasumatsu, 1955: 246. Holotype (male, KUF), type loc.: Sobosan, Prov. Bungo, Kyusyu, Japan. Acrepyrisminutus: Terayama, 1996: 595 (genus transfer). Pristepyrisminutus: Azevedo et al. 2018: 104 (genus transfer). Comb. nov.
Apenesia takasago Terayama, 1996: 143, figs 15–18. Holotype (male, NSMT), type loc.: Tokkasha, Nantou Hsien, Taiwan. Pristepyristakasago: Azevedo et al. 2018: 104 (genus transfer). Syn. nov.

###### Male diagnosis.

TL ≈ 3.3–5.5 mm. HL/HW × 100 = 98–109. Frons and vertex almost smooth and shining or with shallow foveolae, of which intervals are smooth and shining and wider than diameter of foveolae. Anterior clypeal margin nearly straight. Mandible with four apical teeth. Transverse pronotal carina absent. Cervical pronotal area in lateral view gently rounded. LP/WP = 1.30–1.44. Metapostnotal median carina distinct, but incompletely reaching posterior transverse margin. Tergum II without longitudinal ridge and sulcus, sternum II with longitudinal median carina. Hypopygium with almost straight apical margin. Aedeagus with developed ventral and dorsal valves; apical lobe reduced.

###### Female diagnosis.

TL = 3.7 mm. HL/HW × 100 = 139. Frons and vertex with foveolae (ca. 0.01 mm in diameter), of which intervals are imbricate; intervals in lateral part of frons as wide as or narrower than diameter of foveolae; intervals in vertex and median part of frons wider than diameter of foveolate. Median portion of clypeus roundly produced anteriad. Compound eye less developed. Mandible with four teeth. Transverse pronotal carina absent. Cervical pronotal area in lateral view gently rounded. Dorsal area of pronotum, mesoscutellum, mesopleuron and dorsomedian face of metapectal-propodeal complex imbricate. Dorsal area of pronotum, mesoscutellum, mesopleuron and dorsolateral face of metapectal-propodeal complex with spare foveolae. Tergum II without longitudinal ridge and sulcus.

###### Male description.

Full description was given by Yasumatsu (1955) and Terayama (2006). Additional information as below.

***Head*.**HL/HW × 100 = 98–109 (100 in holotype of *P.minuta*). Frons and vertex almost smooth and shining or with inconspicuous foveolae (ca. 0.01–0.02 mm in diameter, Fig. 15A) or shallow foveolae (ca. 0.02–0.04 mm, Fig. 15B), of which intervals are smooth and shining and wider than diameter of foveolae. Occipital carina present. Median portion of clypeus shortly produced anteriad; median clypeal carina moderately distinct, not reaching anterior margin; anterior clypeal margin nearly straight medially. Compound eye large and convex, with sparse thin erect setae. Mandible with four teeth.

***Mesosoma*.** Pronotum without transverse pronotal carina; cervical pronotal area in lateral view round; dorsal area smooth and shining, or with sparse, inconspicuous or shallow foveolae. Mesopleuron elongate; anterior, upper and lower fovea distinct; acropleural area smooth and shining, with inconspicuous foveolae; mesopleural pit absent. Mesodiscrimen concave, without median carina. Metasternum with metafurcal pit. Lateral face of metapectal-propodeal complex obliquely rugose in marginal area and irregularly rugose in central area. Metapectal-propodeal complex in dorsal view with lateral margins subparallel and slightly convex; LP/WP = 1.30–1.44 (1.30 in holotype of *P.minuta*); metapostnotal median carina distinct, but incompletely reaching posterior transverse margin; submedian rugae irregularly running; sublateral margin distinct, but short, incomplete posteriorly; posterior transverse margin distinct; dorsomedian face weakly rugoso-scabrous; dorsolateral face smooth and shining; median portion of propodeal declivity with transversely rugoso-scabrous. Forewing with long R1_2_v vein and R_2_ flexion line, of which the latter is shorter than 1M_2_ flexion line (arrows in Fig. 15F), without 2M1_2_ flexion line. Hindwing with four distal hamuli. Tarsal claws bifid, with thin and curved apical teeth; basal one very short.

***Metasoma*.** Tergum II without longitudinal ridge and sulcus; sternum II with longitudinal median carina. Hypopygium with very long spiculum, with almost straight apical margin; apicomedian part thickened which is visible as a small triangular region; outer face of apicomedian part with relatively dense setae; membrane developed between spiculum and S9ala (Fig. 17A), without thickened margin. Gonostipes thin and glabrous, fused to harpe in dorsal portion. Harpe in ventral view elongate-spatulate, slightly curved inward, with blunt apex, entirely covered with setae which increase in length toward apex; median basal portion with concavity which accommodates digitus and cuspis. Subbasal part of volsella with seta-bearing area which is almost as long as cuspis. Cuspis elongate-lobate and straight, extending posteriad, with several long setae at the apex. Digitus extending laterad, curled. Aedeagus with reduced apical lobe; dorsal lobe large; ventral lobe elongated, with large lobate projection produced ventrally in posterior portion (arrow in Fig. 17D, F, H).

###### Female description.

Female of this species was recognized for the first time by collecting a male and female pair in copulation.

***Color*.** Body light brown; mandible, antenna and legs as same as or lighter than body.

***Head*.** Head capsule with posterior margin very weakly incurved, with posterolateral corner round; HL/HW × 100 = 139. Occipital carina present. Frons and vertex foveolate (ca. 0.01 mm in diameter), with intervals imbricate; intervals in lateral part of frons as wide as or narrower than diameter of foveolae; intervals in vertex and median part of frons as wide as or wider than diameter of foveolae. Clypeus imbricate; median portion roundly produced anteriad; median longitudinal carina reaching anterior clypeal margin which is slightly incurved medially (Fig. 18C). Compound eye less developed. Mandible with four teeth. Antennomere I (excluding the basal condylar bulb) 3.1× as long as maximum width; antennomere I:II:III = 27:8:6 in length; antennomere II 0.9× as long as maximum width, narrowed and bent in basal part; antennomere III–XII each 0.72–0.78× as long as maximum width, elongate-cylindrical; antennomere XIII (terminal) 1.7× as long as maximum width, with round apex.

***Mesosoma*.** Pronotum with anterior flange extending anteriad beyond anterior margin of propleuron; cervical pronotal area in lateral view gently round; dorsal area subtrapezoidal, with weakly incurved posterior margin, with inconspicuous foveolae of which intervals are imbricate and wider than diameter of foveolae; transverse pronotal carina absent; LPD/WPD = 1.36. Mesoscutum overlain by posteromedian portion of pronotum. Mesoscutellum trapezoidal, 0.72× as long as maximum width, weakly imbricate, with a few inconspicuous foveolae. Mesopleuron elongate and imbricate; anterior, upper and lower depressions absent; mesopleural pit absent. Lateral face of metapectal-propodeal complex imbricates entirely. Metapectal-propodeal complex in dorsal view weakly constricted behind propodeal spiracles and then widened again posteriad, without any distinct carinae which subdivide dorsal face; LP/WP = 2.16; dorsomedian face smooth and shining; median portion of propodeal declivity weakly transversely rugoso-scabrous.

***Metasoma*.** Tergum II without longitudinal ridge and sulcus.

###### Material examined.

**Japan: Tokyo.** 4 males (JT200820_01, 05–07); Minami-osawa, 35°37'11"N, 139°12'03"E, 154 m alt. HauChuan Liao leg. (sweeping); 20/VIII/2020. 1 female, Miyake-jima; Kentaro Tsujii leg.; 25/VIII–22/IX/2012. **Yakushima.** 2 males (JK210921_05, 07); Ohko-no-taki, 30°17'48"N, 130°24'51"E, 16 m alt. HauChuan Liao leg. (sweeping); 22/IX/2021. **Okinawa-Hontou.** 1 male (JO170808_05); Mt. Nago, 26°35'58"N, 128°01'09"E, 181 m alt. HauChuan Liao leg. (sweeping); 10/VIII/2017. 2 males (JO190717_13, 15); Kunigami Vil., 26°44'41"N, 128°13'10"E, 316 m alt. HauChuan Liao leg. (sweeping); 19/VII/2019. **Amami-Oshima.** 1 male (JA170808_13); Mt. Yuwan, 28°16'13"N, 129°19'26"E, 44 m alt. HauChuan Liao leg. (sweeping); 16/VIII/2017. **Ishigaki**–**jima.** 3 males (JI170808_28, 31, 35), Mt. Omoto, 24°26'31"N, 124°05'56"E, 93 m alt. HauChuan Liao leg. (sweeping); 12–13/VIII/2017. **Iriomote**–**jima.** 2 males (JIR190717_49, 54), Tropical Biosphere Research Center, 24°23'48"N, 123°48'11"E, 33 m alt. HauChuan Liao leg. (sweeping); 27–28/VII/2019. **Taiwan: N. Taiwan.** 3 males (TNT180629_03, 04, 09), Mt. ShiZaiTou, New Taipei City, 24°54'14"N, 121°29'46"E, 778 m alt. HauChuan Liao leg. (sweeping); 29/VI/2018. 5 males (TNT180706_01, 04, 06–08), Mt. Ta Tung, New Taipei City, 24°52'53"N, 121°34'07"E, 602 m alt. HauChuan Liao leg. (sweeping); 6/VII/2018. **C. Taiwan.** 2 males (TN181022_40, 47); Sun Moon Lake, Nantou County, 23°50'57"N, 120°56'16"E, 92 m alt. HauChuan Liao leg. (sweeping); 23/X/2018.

**Figure 19.**
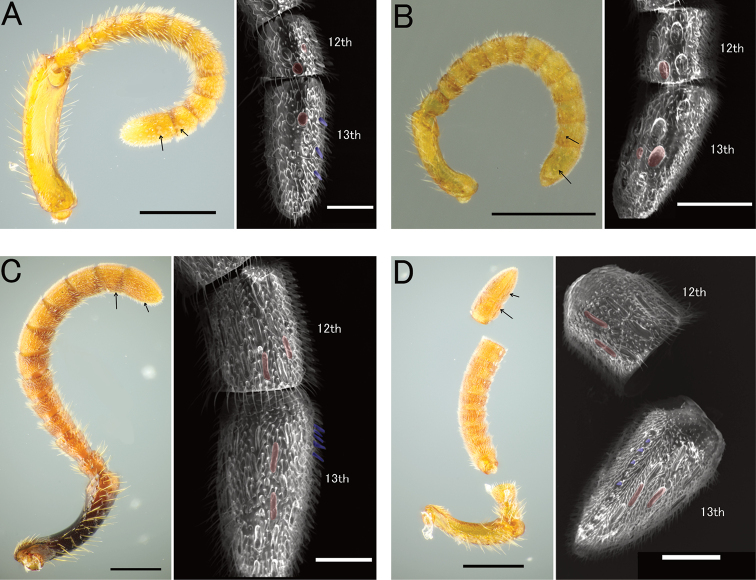
Sensilla placodea (red) and sensilla basiconica (blue) in female wasps, with SEM image. **A***Apenesiamakiharai*, JO180206_01 **B***Eleganesiatakasago*, TP170606_C2 **C***Pristepyriszhejiangensis*, JM090717_28 **D***Propristocera* sp. Scale bars: 0.25 mm in light microscope images; 50 μm in SEM images.

###### Taxonomic remarks.

Due to the new combination of “*Pristepyrisminutus*” to the genus *Elganesia*, the “Key to Taiwanese and Ryukyuan species of the genus *Eleganesia*, based on male morphology” given in Liao et al. (2021) is updated and given as Appendix 1.

The holotype of “*Pristepyristakasago*” was unable to be discriminated morphologically from *E.minuta* (including the holotype). Therefore, the former is herein synonymised under the latter.

In the present phylogenetic tree (Fig. 3), *E.minuta* was subdivided into three (or four) lineages, i.e. Lineage “X” from the Kanto area of Japan to Okinawa-Hontou; “Y” from Ishigaki–jima to Taiwan and “Z” from Taiwan. The lineages also showed differences in the shape of the lobate extension of the ventral valve of aedeagus (Lineage X as in Fig. 17C, D; Y as Fig. 17E, F; Z as Fig. 17G, H). However, there was no remarkable difference among them in external morphology and hypopygium (excluding weak variation in head sculpture as seen in Figs 14A, 15A, B, 16A) and also no differences in the 28S sequence (Table 2). As the lineages were parapatric or allopatric in the area of Taiwan and the Ryukyus, in the present study, the Lineage X, Y and Z are conspecific and treated as *E.minuta*. However, it is also possible that further taxon sampling and integrative taxonomy may reveal several cryptic species within *E.minuta* and determine one of them as “*E.takasago*” (see also “Discussion”).

###### Distribution and habitat.

Hokkaido to Ryukyus in Japan (Terayama 2006), northern South Korea (Lim et al. 2011), northern and central Taiwan; evergreen broadleaf forest.

## Supplementary Material

XML Treatment for Pristepyris seqalu

XML Treatment for Pristepyris ishigakiensis

XML Treatment for Pristepyris mieae

XML Treatment for Pristepyris ryukyuensis

XML Treatment for Pristepyris tainanensis

XML Treatment for Pristepyris zhejiangensis

XML Treatment for Eleganesia minuta

## ﻿Appendix 1

Updated key to Taiwanese and Ryukyuan species of the genus *Eleganesia*, based on male morphology

The following key is partly modified from Liao et al. (2021) in order to involve *E.minuta* comb. nov.

**Table:**

1	Mandible with 4 teeth	**2**
–	Mandible with 5 teeth	**7**
2	Dorsolateral face of metapectal-propodeal complex smooth and shining, inner membrane of hypopygium without anterior margin	***E.minuta* (Yasumatsu, 1955) comb. nov.**
–	Dorsolateral face of metapectal-propodeal complex rugose, inner membrane of hypopygium with anterior margin	**3**
3	Dorsal face of head and dorsal pronotal area with foveolae of which interspaces are imbricate	***E.liukueiensis* (Terayama, 1996)**
–	Dorsal face of head and dorsal of pronotum with foveolae of which interspaces are smooth	**4**
4	Antennomere III to XII short, 2.0× as long as wide	***E.takasago* (Terayama, 1996)**
–	Antennomere III to XII long, more than 2.5× as long as wide	**5**
5	Head long, HL/HW = 121. Compound eye with relatively long erect setae	***E.paiwan* Liao et al., 2021**
–	Head relatively round, HL/HW less than 115. Compound eye with short erect setae	**6**
6	Thickened region of apicomedian part of hypopygium trapezoidal. Ventral valve of aedeagus in lateral view with posteroventral projection quadrate	***E.elegans* (Terayama, 1999)**
–	Thickened region of apicomedian part of hypopygium triangular. Ventral valve of aedeagus in lateral view with posteroventral projection narrowly produced	***E.kijimuna* Liao et al., 2021**
7	Antennomere III to XII 2.0–2.4× as long as wide. Frons and vertex with dense and shallow foveolae of which intervals are imbricate. LP/WP = 1.46–1.60. Apical margin of hypopygium broadly and evenly concave	***E.meifuiae* (Terayama, 1996)**
–	Antennomere III to XII 3.0× as long as wide. Frons and vertex with dense and deep foveolae of which intervals are smooth and shining. LP/WP = 1.30–1.45. Apical margin of hypopygium with a median angular projection	***E.chitouensis* (Terayama, 1996)**
